# High-Energy-Density Organic Amendments Enhance Soil Health

**DOI:** 10.3390/ijerph191912212

**Published:** 2022-09-26

**Authors:** Feifan Shi, Xinyue Zhao, Qilu Cheng, Hui Lin, Huabao Zheng, Qifa Zhou

**Affiliations:** 1 College of Life Sciences, Zhejiang University, Hangzhou 310058, China; 2 Institute of Environment Resources Soil and Fertilizers, Zhejiang Academy of Agricultural Sciences, Hangzhou 310021, China; 3 Zhejiang Province Key Laboratory of Soil Contamination Bioremediation, Zhejiang A&F University, Hangzhou 311300, China

**Keywords:** high-energy-density organic amendments, microbial growth, bacterial:fungal ratio, microbial community, microbe–crop competition, soil health

## Abstract

Soil microbial biomass (SMB) and soil microbial communities (SMCs) are the key factors in soil health and agricultural sustainability. We hypothesized that low bioavailable carbon (C) and energy were the key limiting factors influencing soil microbial growth and developed a new fertilization system to address this: the simultaneous application of mineral fertilizers and high-energy-density organic amendments (HED-OAs). A microcosm soil incubation experiment and a *Brassica rapa* subsp. *chinensis* pot culture experiment were used to test the effects of this new system. Compared to mineral fertilizer application alone, the simultaneous input of fertilizers and vegetable oil (SIFVO) achieved a bacterial abundance, fungal abundance, and fungal:bacterial ratio that were two orders of magnitude higher, significantly higher organic C and nitrogen (N) content, significantly lower N loss, and nearly net-zero N_2_O emissions. We proposed an energy and nutrient threshold theory to explain the observed bacterial and fungal growth characteristics, challenging the previously established C:N ratio determination theory. Furthermore, SIFVO led to microbial community improvements (an increased fungal:bacterial ratio, enriched rhizosphere bacteria and fungi, and reduced N-transformation bacteria) that were beneficial for agricultural sustainability. A low vegetable oil rate (5 g/kg) significantly promoted *Brassica rapa* subsp. *chinensis* growth and decreased the shoot N content by 35%, while a high rate caused severe N deficiency and significantly inhibited growth of the crop, confirming the exceptionally high microbial abundance and indicating severe microbe–crop competition for nutrients in the soil.

## 1. Introduction

Fertilizers play a crucial role in crop production worldwide. However, fertilization can cause pollution and soil degradation, thus reducing agricultural sustainability [1,2,3,4]. Therefore, improvements in nutrient management are required to meet the food security and sustainability challenges of the coming decades [1,2]. N loss mitigation may become the primary goal in improving nutrient management. Soil microbial biomass (SMB) and soil microbial communities (SMCs) significantly influencing soil fertility, and cycles of C and N are the key factors in soil health and agricultural sustainability [5,6,7]. In particular, higher fungal:bacterial ratios indicate more sustainable soil systems [7], and enhancing the SMB of beneficial microorganisms, such as fungi, is a game-changer in restoring soil functions in degraded land [8]. Various management strategies have been employed to enhance the SMB in extensive previous studies [6,9], and the application of organic amendments (OAs) is currently the most effective strategy available. OAs used in agriculture include biosolids, animal manure, municipal solid wastes, yard waste compost, crop residues, seaweed, blood and bone meal, and humic substances and biochars [6,10]. However, previous studies have found that the growth stimulation effect of these OAs appears to be very limited [11,12,13,14,15], even at high application rates and when applied for long periods of time. On a global basis, OAs have been found to increase MBC by an average of 51% more than mineral-only fertilization [11]. Specifically, in nutrient-rich mollisols, incorporating legumes and manure into annual cropping systems enhanced the SMB by nearly 200% [14], while the application of different composts at a rate equivalent to 175 kg N ha^−1^ yr^−1^ for 12 years led to an increase in MBC of less than 50% [13]. The weak response of SMB to OAs could be attributed to the low bioavailable C and energy density in the OAs, because both energy and C sources are essential for sustaining microbial growth. Therefore, it was hypothesized that amending soil with high-energy-density (HED)-OAs could substantially stimulate microbial growth and profoundly reshape the microbial structure, thus altering C and N processes in soil. It was further hypothesized that HED-OAs could increase the fungal:bacterial ratio, because fungi require more energy and C than bacteria. Vegetable oil (VO) is a well-known substance that has a high bioavailable energy density and C content because it is mainly composed of high-energy storage lipids, such as triacylglycerol (TAG). Unlike other solid OAs, VO can be easily and thoroughly mixed with soil, enabling the uniform distribution of energy and nutrients, such as C, and thus favoring microbial growth and microbial biomass production throughout the soil. Rapid microbial growth linked to the rapid assimilation of inorganic N into organic N can mitigate N loss and N_2_O emissions, which are of great concern [4,10]. This study explored the potential ability of SIFVO to enhance soil health and agricultural sustainability by investigating the effects of SIFVO on microbial growth and structure, soil C and N contents, N loss, greenhouse gas (GHG) emissions, and crop growth.

## 2. Materials and Methods

### 2.1. Experimental Design

A mesocosm incubation (MI) experiment was performed by incubating 250 g soil in a 660 mL glass bottle from November to December 2021. The sandy loam soil was an inceptisol obtained from 0 to 5 cm surface soil on a vegetable farm in Zhejiang University, Hangzhou, China. The soil was air-dried and passed through a 2 mm sieve before use. The soil properties are shown in Table S1. There were four treatments: (1) control (CK); (2) mineral fertilizer input alone (Fa), with the application of KH_2_PO4 (1.740 g/kg), urea (1.488 g/kg), and (NH_4_)_2_SO4 (0.336 g/kg); (3) vegetable oil input alone (VOa), with the application of VO at rates of 5, 10, 20, and 40 g/kg; and (4) the simultaneous input of the same fertilizers and VO as in Fa and VOa (SIFVO). The fertilizer rates were designed according to our preliminary test results. An additional VO rate (2 g/kg) in the SIFVO treatment was used for macroscopic observation. The VO used had a C content of 66.2% and was a 4:0 fatty acid-dominant (98.2%) commercial product (Table S2). There were three replications for each treatment. The soil was initially moistened with 75 mL of ultra-pure water and incubated for 48 days at a temperature of 25 °C and relative humidity (RH) of 70%. A pot culture (PC) experiment was conducted from November to December 2021 in a greenhouse at the Experimental Farm of Zhejiang University, China. The light conditions were natural throughout the entire culture period. Temperature was controlled (a daily maximum of 30.5 °C, and a daily minimum of 20.0 °C), and the average daily RH ranged from 50.0% to 85.5%. The soil was the same as that used in the MI experiment. The treatments consisted of the same Fa and FVO treatments with three replications in the MI experiment. Each 1 L plastic pot was filled with 500 g of soil. A popular vegetable crop in China, *Brassica rapa* subsp. *chinensis* cv. “Dongchangqin” was seeded in each pot, and the most vigorous seedling was retained in each pot after germination. The soil was watered every two days.

### 2.2. Soil Property Measurement

Soil EC was measured with a EC meter (DDSJ-308F, Leichi, Shanghai, China) via mixing 3.00 g soil with 3.00 mL ultra-pure water. Soil C and N contents were determined using finely ground (<0.150 mm) subsamples using a vario isotope cube analyzer (Elementar, Hanau, Germany). Soil inorganic C was removed by acidification with HCl during the instrumental analysis. Soil nitrate nitrogen and ammonia nitrogen contents were measured with a flow continuous analyzer (SKALAR/SAN++, SKALAR, Netherlands) after mixing 3.00 g of fresh soil with 5.00 mL 2 M KCl solution and then centrifuging at 600 rpm to sample the supernatant. Soil GHG (N_2_O, CH_4_, and CO_2_) fluxes were determined on incubation day 8, 24, and 48 for the microcosm bottles. To ensure the same initial gaseous concentrations at the start of the incubation period during sampling, ambient air was flushed into each bottle for 2 min. Bottles were then sealed with parafilm for 3 h to accumulate GHGs in the headspace. A gas sample was then collected for quantification of GHGs using gas chromatography (GC-200 Plus, Shimadzu, Kyoto, Japan).

### 2.3. Soil DNA Extraction and High-Throughput Sequencing

Five grams of soil from each bottle/pot were sampled for analysis. A TIANNAMP Soil DNA Kit (TIANGEN, Beijing, China) was used to extract soil DNA. The quantity and quality of DNA were evaluated, and the DNA was then stored at −80 °C before use. Sample integrity was tested by agarose gel electrophoresis. The 16S rRNA gene of bacteria (in the V3–V4 region) was amplified using the primers 515F and 806R, and 528F and 706R. The rRNA gene of fungi was amplified using the ITS1-F, ITS2-2043R, and ITS5-1737F primers. The sequencing of bacteria and fungi was performed using the Illumina HiSeq 2500 platform (BGI Co., Ltd., Shenzhen, China). Sequence analyses were conducted according to the methods of Yan et al. (2022) [16].

The 16 S rRNA gene and the ITS gene were quantified using a StepOnePlus™ Real-Time PCR System (Thermo Fisher, Waltham, MA, USA) and TB Green™ Premix Ex Taq™ II (Tli RNaseH Plus) kit (Takara, Kyoto Japan). The qPCR conditions, primer sequences targeting these genes, and PCR protocol were based on previously reported methods [17]. Each reaction contained 0.8 µL of primers, 1 µL of DNA templates, 5 µL of TB Green Premix Ex Taq II (Takara, Japan), 0.2 µL of ROX Reference Dye (Bio-Rad), and 3 µL of ddH_2_O (Bio-Rad). Each PCR reaction was conducted in triplicate.

### 2.4. Plant Analysis

The crop was harvested 48 days after seeding. The crop was weighed to obtain the fresh mass and then oven-dried at 70 °C and weighed to determine the root dry mass and shoot dry mass. The dry samples were finely ground into powder (particle sizes <0.150 mm) and then analyzed to determine the C and N contents using a vario isotope cube analyzer (Elementar, Hanau, Germany). The fatty acid composition in the VO was determined according to PRC National Standard Method GB/T17736-2008.

### 2.5. Data Analysis

Tukey tests were used to compare means with 0.05 set as the significance level. Soil C (N) loss was calculated as the difference in C (N) content between the tested soil and the initial fertilized soil in the MI experiment, and soil N loss was calculated as the initial fertilized soil N content plus crop N accumulation (root dry mass × root N content + shoot dry mass × shoot N content), subtracting the tested soil N content. The relative abundance for each taxon was calculated as the operational taxonomic unit (OTU) ratio (%) among the total OTUs. The relationship between the bacterial or fungal relative abundance and the VO rate was fitted with an exponential equation, and the coefficient of determination (R^2^) was employed to assess the sensitivity of bacteria and fungi to VO. For convenience, the SIFVO treatments at VO rates of 0, 5, 10, 20, and 40 g/kg were designated as SIFVO0, SIFVO5, SIFVO10, SIFVO20, and SIFVO40, respectively, and SIFVO0 was the same treatment as Fa.

## 3. Results and Discussions

### 3.1. Soil Health Indicators and N Loss under Different VO Application Rates

As shown in Table 1, soil organic C (SOC) and soil total N (STN) increased with the VO application rate, and soil N loss decreased while soil C loss increased with rising VO application rates. The differences among the SIFVO treatments were mostly significant (*p* < 0.05) and consistent for these indicators. Soil electrical conductivity (EC) (Figure S1a), NH_4_-N (Figure S1b), and NO_3_-N (Figure S1c) consistently and significantly (*p* < 0.05) decreased with the increasing VO rate. Notably, the NO_3_-N content in the SIFVO0 (Fa) treatment remained high the entire incubation period. The soil pH was lower in SIFVO0 than in SIFVO5 and decreased with the VO rates from 5 g/kg to 40 g/kg (Figure S1d). The higher STN and lower EC and inorganic N contents in the higher VO treatments clearly indicated that inorganic salts (e.g., inorganic N, phosphate, sulfate, and K^+^) incorporated by microbes increased with the VO application rate, because more than 80% of the soil organic N consisted of microbial N [18]. As NH_4_-N was the dominant ionic species in the soil, microbial uptake of NH_4_-N could acidify the soil, which could explain that the soil pH decreased with the VO rates from 5 g/kg to 40 g/kg. The soil pH was lower in SIFVO0 than in SIFVO5, which might be attributed to the stronger nitrification–dinitrification activity in SIFVO0. The CO_2_ flux increased consistently and significantly (*p* < 0.05) with the increasing VO rate (Figure S2a). The CH_4_ fluxes were very low for all treatments on day 8 and increased consistently and significantly (*p* < 0.05) from 0 to 20 g/kg, and then decreased on days 24 and 48 (Figure S2b). The N_2_O flux drastically decreased with the increasing VO rate, although the SIFVO5 treatment had a higher N_2_O flux than the N_2_O fluxes of the Fa and SIFVO40 treatments on day 8. The N_2_O fluxes of the SIFVO5, SIVO10, SIFVO20, and SIFVO40 treatments on days 24 and 48 were close to net zero (Figure S2c). The bacterial abundance, the fungal abundance, and the fungal:bacterial ratio (Table 1) tended to increase with the VO rates, although the fungal abundance and the fungal:bacterial ratio tended to decrease when the VO rate was above 10 g/kg on day 48 (Table 1). Compared to the Fa treatment, the SIFVO treatments (SIFVO5, SIFVO10, SIFVO20, and SIFVO40) increased the bacterial abundance, fungal abundance, fungal:bacterial ratio, soil organic C content, soil N content, and C loss by averages of 1042%, 26182%, 2334%, 129%, 24%, and 273%, respectively, and mitigated N loss by 46%. Additionally, the N losses for the SIFVO treatments on day 48 were significantly lower in the pot culture (PC) experiment than in the mesocosm incubation (MI) experiment owing to crop N utilization (Table 1). SIFVO treatment mitigated N_2_O flux by −74.1–97.9%, 96.9–99.2%, and 97.4–99.8% on days 8, 24, and 48, respectively, compared to Fa. Consistently, the C loss, the CO_2_ flux, and the CH_4_ flux were higher in the SIFVO treatments than in the Fa treatment (Table 1).

### 3.2. Microbial Responses to the Input of Fertilizers and VO

**Figure 1:**
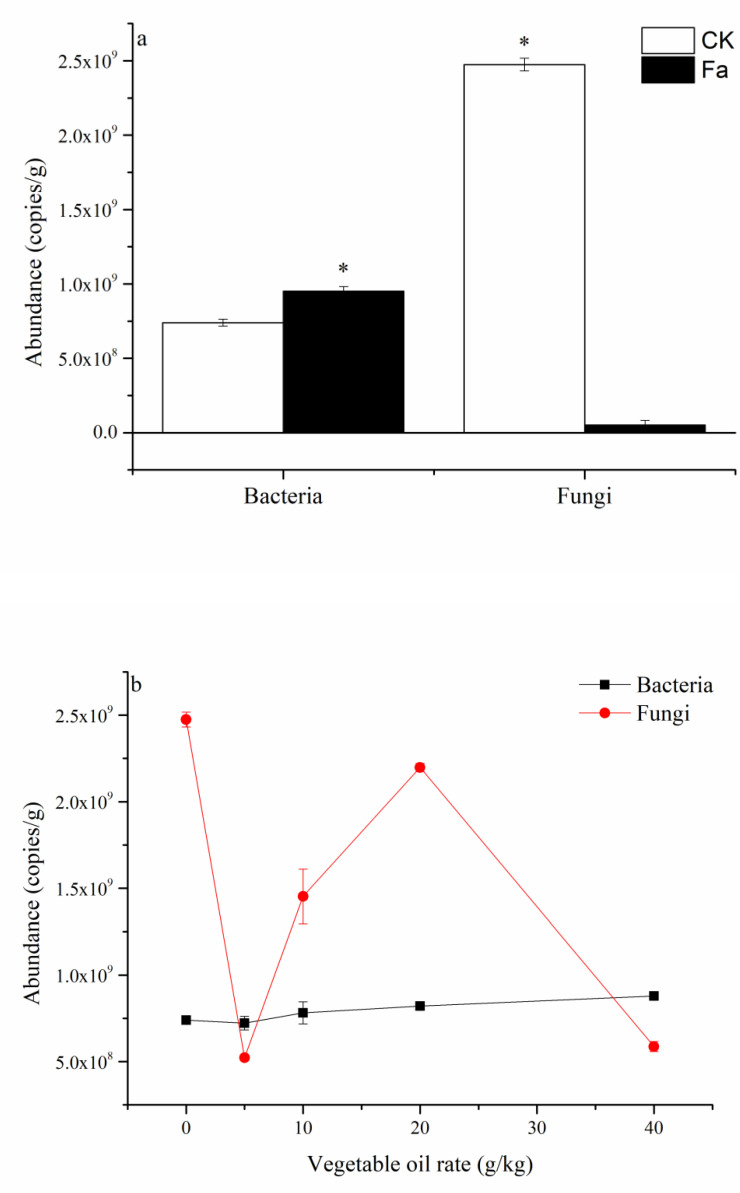
Soil bacterial and fungal abundance in the control (CK), fertilizer input alone (Fa), and vegetable oil alone (VOa): (**a**) CK and Fa on day 8, (**b**) VOa on day 8. Data shown are the means of three replicates, and * indicates significance at 0.05.

**Figure 2:**
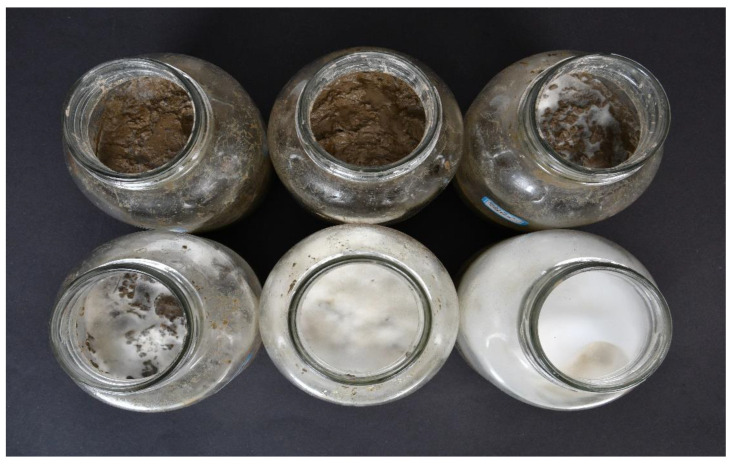
Fertilizer and vegetable oil (SIFVO) treatments on day 4 in the mesocosm incubation (MI) experiment. The bottles from upper left to the lower right were treated with the same fertilizers and 0, 2, 5, 10, 20, and 40 g/kg of vegetable oil.

As compared to the CK on day 8 in the MI experiment, Fa and VOa led to increases in bacterial abundance of 5.7% and −2.3–18.8%, respectively, and significant (*p* < 0.05) decreases in fungal abundance of 96.3% and 11.1–78.8%, respectively (Figure 1). SIFVO application greatly increased the fungal abundance as the fungal mass on the soil surface increased with the VO application rate (Figure 2). Compared to Fa, SIFVO treatment increased bacterial abundance by 668.4–1415.7%, 8.97–1528.2%, 912.9–3185.7%, and 264.7–576.5% on days 8, 24, and 48 in the MI experiment and day 48 in the PC experiment, respectively, and increased fungal abundance (fungal:bacterial ratio) by 13361.5–42015.3% (1696.2–2714.8%), 1810.1–61360.6% (1718.3–5763.6)%, 23.6–75872.2% (−88.2–4727.9%), and 7400.0–11400.0% (1040.0–3120.0%) on days 8, 24, and 48 in the MI experiment and day 48 in the PC experiment, respectively (Table 1). The only negative increase in the fungal:bacterial ratio occurred in the SIFVO5 treatment incubated for a long period of time (48 days). Therefore, the growth stimulation efficiency in the SIFVO treatments could be two orders of magnitude higher than found in previous studies [6,9,11,13,14]. Fa led to a limited increase in bacterial abundance, which was in agreement with the findings of previous studies [19]. The application of VO alone led to a limited increase in bacterial abundance, which could be explained by the fact that nutrients limited the bacterial growth [18]. Numerous studies support the C:N ratio determination theory, which proposes that fungal biomass decreases and bacterial biomass increases with decreasing soil C:N ratio [16,20,21,22,23,24]. However, in the present study, it was observed that both fertilizer input alone, which decreased the C:N ratio, and VO input alone, which increased the C:N ratio, increased the bacterial abundance and decreased the fungal abundance (Figure 1). In the MI experiment, the bacterial abundance tended to increase with the soil C:N ratio from 7.3 to 18, and then it began to decrease, and the link between fungal abundance and the soil C:N ratio was weak (Figure S3). Thus, an energy and nutrient threshold theory can be proposed to explain the observed fungal growth characteristics.

The energy and nutrient threshold theory developed in this study states that the soil fungal community can only maintain growth when both energy levels and nutrient levels are above threshold values, and threshold values change with the growth or decline of the bacterial community and fungal community. Fertilizer input and VO input in energy- and nutrient-poor soil could promote bacterial growth and thus decrease the energy level or nutrient level to below the thresholds for fungal growth, thus decreasing fungal abundance (Figure 1). When the fungal abundance decreased to a certain level, decreasing the threshold values, fungi could begin a new cycle of growth, and the fungal abundance increased as the VO rate increased from 0 to 20 g/kg and then decreased due to nutrient (in this case, N) limitation in the 40 g/kg treatment caused by the rapid consumption of nutrients by growing bacteria and fungi. The lower fungal abundance and fungal:bacterial ratio at high VO rates (20 and 40 g/kg) (Table 1) could also be attributed to nutrient (N) limitation (Figure S1) on fungal growth.

At the phylum level, Proteobacteria was the most dominant bacteria in all the treatments, and Firmicutes was enriched and Bacteroidota reduced in the VO treatments compared to the treatments without OA in the MI experiment and PC experiment (Figure S4). Mortierellomycota and Ascomycota were dominant, as the other fungal phyla were extremely low in abundance in all treatments (Figure S5). Very interestingly, the most dominant bacterial genera (TBG1) were N-transformation species including *Flavisolibacter* spp. (involved in N removal [25]), *Solitalea* spp. (involved in ammonia oxidation [26]), *Castellaniella* spp. (involved in denitrifyication [27]), and *Herbaspirillum* spp. (diazotrophic bacteria [28]) in the Fa treatments (Figure S6a), while the TPG1 included rhizosphere bacterial species (*Acinetobacter* spp. [29], *Sphingobacterium* spp. [30], *Simplicispira* spp. [31], *Chitinophaga* spp. [32], *Noviherbaspirillum* spp. [33], and *Sphingopyxis* spp. [30]), lipid-digestion bacteria (NK4A214_group [34] and *Syntrophomonas* spp. [35]), and magnetotactic bacteria (*Magnetospirillum* spp. [36]) (Figure S6a). The TPG1 for the VOa treatments included rhizosphere bacteria (*Massilia* spp. [33] and *Cellvibrio* spp. [35]) and sulfate-reducing bacteria (*Pelosinus* spp. [37]) in the VO treatments (Figure S6a). The most dominant fungal genera (TFG1) included the thermophilic *Thermomyces* spp. [38] in the Fa and the CK treatments, and rhizosphere fungal species (*Malassezia* spp. [31], *Scutellinia* spp. [32], *Mortierella* spp. [39], and *Mortierellales* spp. [39]), pathogenic species (*Corallomycetella* spp. [40]), and species that were not clearly characterized (*Cheilymenia* spp. and *Allomyces* spp.) in the VO treatments (Figure S6b). Additionally, analysis of the microbial relative abundance trends (Figures S7 and S8) revealed that some key N-transformation bacteria, including *Flavisolibacter* spp. (Figure S7a), *Solitalea* spp. (Figure S7b), *Herbaspirillum* spp. (Figure S7h), and well-known ammonia oxidation bacteria (*Nitrosospira* spp. and *Nitrosomonas* spp.) (Figure S7d,e), as well as some fungal species (including *Thermomyces* spp.) (Figure S8a), were inhibited by VO, while some rhizosphere bacteria, including *Acinetobacter* spp. (Figure S7i) and *Chitinophaga* spp. (Figure S7k), in addition to rhizosphere fungi species, including *Mortierella* spp. (Figure S8b) and *Scutellinia* spp. (Figure S8c), were evidently enhanced by VO. *Castellaniella* spp. (Figure S7c) and *Simplicispira* spp. (Figure S7j) seemed to be inhibited by VO. Of particular interest, the relative abundance trend for *Nitrosospira* spp. (Figure S7d) was highly consistent with the N_2_O flux course, and an abundance close to zero was reached concurrently with the nearly net-zero N_2_O emissions (Figure S1c), indicating a determinant role of *Nitrosospira* in N_2_O production. The results support the possibility that *Nitrosospira* is the main genus governing N_2_O production in soils [41]. *Nitrosospira* contains well-known slow-growing species, and slow-growing species may be at a competitive disadvantage under the energy- and nutrient-sufficient conditions found in the SIFVO treatments. *Methanocella* spp. and *Methylobacillus* spp. were the two most abundant methanogens (Figure S7f,g). The relative abundances of the methanogens were very low on day 8, which could explain why the CH_4_ flux was very low under all the VO levels on day 8 (Figure S1b). *Methanocella* and *Methylobacillus* species appeared to be insensitive to VO.

It was reasonable that N-transformation species were dominant in the N-sufficient and energy-poor Fa treatments, and N-assimilation species (mainly rhizosphere bacteria and fungi) were dominant in the N-sufficient and energy-high SIFVO treatments. VO input increased the bioavailable energy and C level and enhanced N assimilation, thus inhibiting the growth of N transformation species and mitigating N loss. As rhizosphere bacteria and fungi are nutrient-efficient [32,33,39], VO input shifted the microbial community from N-transformation-dominant toward N-assimilation-dominant. Consequently, the SOC and STN increased with the VO rate as VO provided bioavailable energy and C, enhanced microbial C and N assimilation, and mitigated N loss. Reasonably, soil C loss increased with the VO rate as the VO input increased the CO_2_ and CH_4_ fluxes by increasing both C substrate and microbial abundance.

### 3.3. Crop Responses

As shown in Figure S9, the crop biomass was significantly higher in SIFVO5 than in SIFVO0 (Fa), and significantly lower in SIFVO10, SIFVO20, and SIFVO40 than in SIFVO0. Additionally, the crop shoot N content decreased consistently with the VO level. The crops exhibited severe N deficiency with stunted growth, yellowed leaves, and very low shoot N content (<2.2%) in the SIFVO20 and SIFVO40 treatments. The significant decrease in crop biomass from SIFVO5 to SIFVO40 could be mainly attributed to the decrease of N available for the crops caused by severe nutrient competition from microbes. In treatment SIFVO5, the microbe–crop nutrient competition was less severe due to the relatively low microbial abundance, and the enrichment of rhizosphere bacteria and fungi could promote crop growth. Therefore, the plant biomass in SIFVO5 was the highest among the five treatments (Figure S9). However, the crop shoot N content decreased by about 35% in SIFVO5 as compared to SIFVO0, indicating that inhibition on crop was still considerable at 5 g/kg of VO. Therefore, a VO rate lower than 5 g/kg should be recommended for crop production.

The current intensification of agricultural practices is already resulting in the unsustainable degradation of soils, mainly including the loss of organic matter, the release of greenhouse gases, and the over-application of fertilizers [42]. N fertilization has substantial adverse impacts on soils and the broader environment, including water pollution from N leaching [4,42], air pollution from the release of nitrous oxide and NH_3_ [4], and soil degradation [4]. The results in this study demonstrated that SIFVO could enhance soil health, thus preventing soil degradation from fertilization. SIFVO could minimize environmental pollution from fertilization, as rapid incorporation of nutrients including N and inhibition of N transformation could substantially mitigate N loss from leaching [4], NH_3_ volatilization [4] and N_2_O emission [4]. Although SIFVO could increase economical costs and carbon emission, this new fertilization system could still achieve the benefits (e.g., healthy soil for sustainably high productivity and minimized pollution), far outweighing the losses. Additionally, use of waste vegetable oil could be economically feasible. However, precision application of VO is needed, as high a VO rate could lead to high C loss, inhibition of crop growth, and enrichment of pathogenic species.

## 4. Conclusions

Compared to CK, Fa, and VOa, SIFVO achieved a bacterial abundance, fungal abundance, and fungal:bacterial ratio that were two orders of magnitude higher. Compared to Fa, SIFVO led to significantly higher organic C and nitrogen (N) content, significantly lower N loss, and nearly net-zero N_2_O emissions, as well as microbial community improvements (an increased fungal:bacterial ratio, enriched rhizosphere bacteria and fungi, and reduced N-transformation bacteria). It was also found that a high VO rate caused severe N deficiency and significantly inhibited growth of the crop, confirming the exceptionally high microbial abundance and indicating severe microbe–crop competition for nutrients in the soil. The results clearly indicated that SIFVO could significantly enhance soil health. However, it was not clear that SIFVO could enhance crop growth.

## Figures and Tables

**Table 1:** Soil health indicators of the simultaneous application of fertilizer and vegetable oil (SIFVO) treatments under different VO application rates in the mesocosm incubation (MI) experiment and the pot culture (PC) experiment.

	VO (g/kg)	SOC (mg/kg)	STN (mg/kg)	BA (10^10^ copies/g)	FA (10^10^ copies/g)	FA/BA	C Loss (mg/kg)	N Loss (mg/kg)
**MI-8**	0	7355 ± 90 a	950 ± 21 a	0.095 ± 0.01 a	0.0052 ± 0.00 a	0.054 ± 0.03 a	2851 ± 90 a	323 ± 21 e
	5	9357 ± 418 b	1007 ± 17 b	0.72 ± 0.10 b	0.082 ± 0.01 b	0.11 ± 0.02 b	2387 ± 500 b	265 ± 18 d
	10	11,210 ± 650 c	1182 ± 22 c	0.73 ± 0.15 b	0.70 ± 0.05 c	0.97 ± 0.15 c	3092 ± 704 c	91 ± 23 c
	20	16,933 ± 611 d	1221 ± 16 d	1.44 ± 0.13 d	2.19 ± 0.05 e	1.52 ± 0.10 e	3166 ± 611 d	51 ± 6 b
	40	30,165 ± 1822 e	1242 ± 15 e	1.24 ± 0.08 c	1.69 ± 0.08 d	1.36 ± 0.05 d	3775 ± 182 e	31 ± 14 a
**MI-24**	0	6932 ± 135 a	909 ± 27 a	0.078 ± 0.01 a	0.0089 ± 0.00 a	0.11 ± 0.01 a	707 ± 135 a	364 ± 28 d
	5	9200 ± 543 b	865 ± 27 a	0.085 ± 0.00 b	0.17 ± 0.00 b	2.00 ± 0.14 b	2545 ± 581 b	407 ± 17 d
	10	11,240 ± 885 c	995 ± 56 b	0.48 ± 0.02 c	1.56 ± 0.17 c	3.18 ± 0.24 c	3062 ± 731 c	277 ± 59 c
	20	17,260 ± 1621 d	1100 ± 45 b	1.27 ± 0.18 e	4.55 ± 0.41 d	3.58 ± 0.18 d	2840 ± 163 c	172 ± 47 b
	40	27,690 ± 2230 e	1195 ± 37 c	0.85 ± 0.11 d	5.47 ± 0.05 e	6.45 ± 0.73 e	6250 ± 223 d	78 ± 38 a
**MI-48**	0	5833 ± 55 a	715 ± 21 a	0.077 ± 0.01 a	0.0072 ± 0.00 a	0.093 ± 0.00 b	1807 ± a	558 ± 22 d
	5	8466 ± 65 b	733 ± 12 a	0.78 ± 0.09 b	0.0089 ± 0.00 b	0.011 ± 0.00 a	3279 ± 55 b	539 ± 12 d
	10	9366 ± 64 c	852 ± 9 b	1.24 ± 0.11 c	5.47 ± 0.49 d	4.49 ± 0.04 e	4936 ± 89 c	421 ± 8 c
	20	15,760 ± 658 d	939 ± 11 c	2.53 ± 0.13 d	1.66 ± 0.03 c	0.66 ± 0.11 c	3255 ± 155 b	333 ± 11 b
	40	21,902 ± 409 e	1041 ± 20 d	1.26 ± 0.02 c	1.56 ± 0.09 c	1.26 ± 0.01 d	12,038 ± 2235 d	232 ± 17 a
**PC-48**	0	6973 ± 90 a	750 ± 21 a	0.34 ± 0.14 a	0.052 ± 0.00 a	0.15 ± 0.08 a	666 ± 41 a	296 ± 30 e
	5	10,306 ± 500 b	836 ± 18 b	1.56 ± 0.10 c	3.20 ± 0.15 b	2.06 ± 0.19 b	1273 ± 195 b	257 ± 23 d
	10	10,612 ± 764 b	1043 ± 23 c	1.24 ± 0.01 b	5.98 ± 0.16 e	4.83 ± 0.06 e	3778 ± 99 c	128 ± 55 c
	20	15,233 ± 612 c	1076 ± 16 c	2.00 ± 0.10 d	4.88 ± 0.20 d	2.44 ± 0.15 d	4866 ± 629 d	83 ± 14 b
	40	22,746 ± 1822 d	1200 ± 14 d	2.30 ± 0.10 e	3.90 ± 0.21 c	1.71 ± 0.13 c	11,193 ± 1844 e	45 ± 15 a

MI-8, MI-24, and MI-48 represent the treatments on days 8, 24, and 48 in the MI experiment, respectively, while PC-48 represents the treatment on day 48 in the PC experiment. SOC: soil organic carbon; STN: soil total nitrogen; BA: bacterial abundance; FA: fungal abundance; FA/BA: fungal:bacterial ratio; C loss: carbon loss; N loss: nitrogen loss. Data are the means ± SD of three replications, and different letters indicate significant differences at 0.05.

## Data Availability

The authors declare that all data and materials are available to be shared upon a formal request.

## Supplementary Materials

The following supporting information can be downloaded at: https://www.mdpi.com/article/10.3390/ijerph191912212/s1, Figure S1: Trends of soil electrical conductivity (a), NH_4_-N (b), NO_3_-N (c) and pH (d) in the fertilizer and vegetable oil (FV-O) treatments during the mesocosm incubation (MI) experiment. Data shown are the means of three replicates, Figure S2: Soil greenhouse gas (GHG) fluxes in the fertilizer and vegetable oil (FV-O) treatments in mesocosm incubation (MI) experiment. (a) CO_2_ flux, (b) CH_4_ flux, and (c) N_2_O flux.Data shown are the means of three replicates, Figure S3: Responses of bacterial and fungal abundance to the soil C:N ratio in the mesocosm incubation (MI) experiment, Figure S4: Bacterial communities at the phylum level for the treatments with the simultaneous application of fertilizer and vegetable oil (SIFVO) and the application of vegetable oil alone (VOa).V.Oa0.8.MI, V.Oa5.8.MI, V.Oa10.8.MI, V.Oa20.8.MI, and V.Oa40.8.MI represent VOa treatment with 0, 5, 10, 20, and 40 g/kg of vegetable oil on day 8 in the mesocosm incubation (MI) experiment, respectively. FV.O0.8.MI, FV.O5.8.MI, FV.O10.8.MI, FV.O20.8.MI, and FV.O40.8.MI represent SIFVO treatment with 0, 5, 10, 20, and 40 g/kg of vegetable oil on day 8 in the MI experiment, respectively. FV.O0.24.MI, FV.O5.24.MI, FV.O10.24.MI, FV.O20.24.MI, and FV.O40.24.MI represent SIFVO treatment with 0, 5, 10, 20, and 40 g/kg of vegetable oil on day 24 in the MI experiment, respectively. FV.O0.48.MI, FV.O5.48.MI, FV.O10.48.MI, FV.O20.48.MI, and FV.O40.48.MI represent SIFVO treatment with 0, 5, 10, 20, and 40 g/kg of vegetable oil on day 48 in the MI experiment, respectively. FV.O0.48.MI, FV.O5.48.PC, FV.O10.48.PC, FV.O20.48.PC, and FV.O40.48.PC represent SIFVO treatment with 0, 5, 10, 20, and 40 g/kg of vegetable oil on day 48 in the pot culture (PC) experiment respectively, Figure S5: Fungal communities at the phylum level for the treatments with the simultaneous application of fertilizer and vegetable oil (SIFVO) and the application of vegetable oil alone (VOa).V.Oa0.8.MI, V.Oa5.8.MI, V.Oa10.8.MI, V.Oa20.8.MI, and V.Oa40.8.MI represent VOa treatment with 0, 5, 10, 20, and 40 g/kg of vegetable oil on day 8 in the mesocosm incubation (MI) experiment, respectively. FV.O0.8.MI, FV.O5.8.MI, FV.O10.8.MI, FV.O20.8.MI, and FV.O40.8.MI represent SIFVO treatment with 0, 5, 10, 20, and 40 g/kg of vegetable oil on day 8 in the MI experiment, respectively. FV.O0.24.MI, FV.O5.24.MI, FV.O10.24.MI, FV.O20.24.MI, and FV.O40.24.MI represent SIFVO treatment with 0, 5, 10, 20, and 40 g/kg of vegetable oil on day 24 in the MI experiment, respectively. FV.O0.48.MI, FV.O5.48.MI, FV.O10.48.MI, FV.O20.48.MI, and FV.O40.48.MI represent SIFVO treatment with 0, 5, 10, 20, and 40 g/kg of vegetable oil on day 48 in the MI experiment, respectively. FV.O0.48.MI, FV.O5.48.PC, FV.O10.48.PC, FV.O20.48.PC, and FV.O40.48.PC represent SIFVO treatment with 0, 5, 10, 20, and 40 g/kg of vegetable oil on day 48 in the pot culture (PC) experiment respectively, Figure S6: Relative abundances of the top bacterial genera (a) and the top fungal genera (b) under different vegetable oil application rate.V.Oa.8.MI, FV.O.8.MI, FV.O.24.MI, FV.O.48.MI, and FV.O.48.PC represent the treatments with vegetable oil application alone on day 8 in the mesocosm incubation (MI) experiment. FV.O.8.MI, FV.O.24.MI, and FV.O.48.MI represent the simultaneous application of fertilizer and vegetable oil treatments on days 8, 24, and 48 in the MI experiment, respectively. FV.O.48.PC represents the simultaneous application of fertilizer and vegetable oil treatments on day 48 in the pot culture (PC) experiment, Figure S7: Responses of the relative abundances of key bacterial taxa to the vegetable oil application rate. The number labeled on the symbol represents the coefficient of determination (R2) in the fitted exponential equation. The unlabeled curves were insignificant in fitting the relationship between the relative abundance and the vegetable oil application rate, Figure S8: Responses of the relative abundances of key fungal taxa to vegetable oil application rates. The number labeled on the symbol represents the coefficient of determination (R2) in the fitted exponential equation. The unlabeled curves were insignificant in fitting the relationship between the relative abundance and the vegetable oil application rate, Figure S9: Crop biomass and the shoot N content under different vegetable oil application rates in the pot culture (PC) experiment.Data shown are the means of three replicates, and different letter indicates significant at 0.05; Table S1: Properties of soil used in this study. Data are the means±Sd of three replications.EC: electrical conductivity; SOC: soil organic carbon; STN: soil total nitrogen; NH_4_-N: ammonia nitrogen; NO_3_-N: nitrate nitrogen, Table S2: Fatty acid (FA) composition of the vegetable oil used in this study.
