# Supplementary material for: High-Energy-Density Organic Amendments Enhance Soil Health
Source: Int J Environ Res Public Health. 2022 Sep 26;19(19):12212. doi: 10.3390/ijerph191912212 (PMC9566092; doi:10.3390/ijerph191912212)
Supplement: Supplementary file 1 [file ijerph-19-12212-s001.zip › SI-R1-Fertilization-zqf.pdf]

Supplementary Information

High-energy-density organic amendments enhance soil health

Shi Feifan[1], Zhao Xinyue[1], Cheng Qilu[2], Zheng Huabao[3]*, Lin Hui[2]*, Zhou Qifa[1]*

[1] College of Life Sciences, Zhejiang University, Hangzhou 310058, China

[2] Institute of Environment Resources Soil and Fertilizers, Zhejiang Academy of Agricultural Sciences, Hangzhou 310021, China

[3] Zhejiang Province Key Laboratory of Soil Contamination Bioremediation, Zhejiang A&F University, Hangzhou 311300, China

* Correspondence: lin82774872@163.com (Lin Hui), zhenghuabao@zafu.edu.cn (Huabao Zheng), zzzqqq@zju.edu.cn (Qifa Zhou).

Table S1. Properties of soil used in this study. Data are the means ± Sd of three replications.EC: electrical conductivity; SOC: soil organic carbon; STN: soil total nitrogen;NH_4-N: ammonia nitrogen;NO_3-N: nitrate nitrogen.

pH	EC (μS/cm)	SOC (%)	STN (%)	NH_4-N (mg/kg)	NO_3-N (mg/kg)
8.00±0.02	146.8±7.7	0.76±0.02	0.057±0.002	3.09±0.09	32.33±0.25

Table S2. Fatty acid (FA) composition of the vegetable oil used in this study

FA	4:0	14:0	15:0	16:0	16:1	17:0	17:1	18:2 EE	18:2 ZZ	18:3 9,12,15, Z	20:3	20:5
%	98.2	0.002	0.0003	0.20	0.002	0.001	0.0006	0.67	0.83	0.01	0.02	0.009

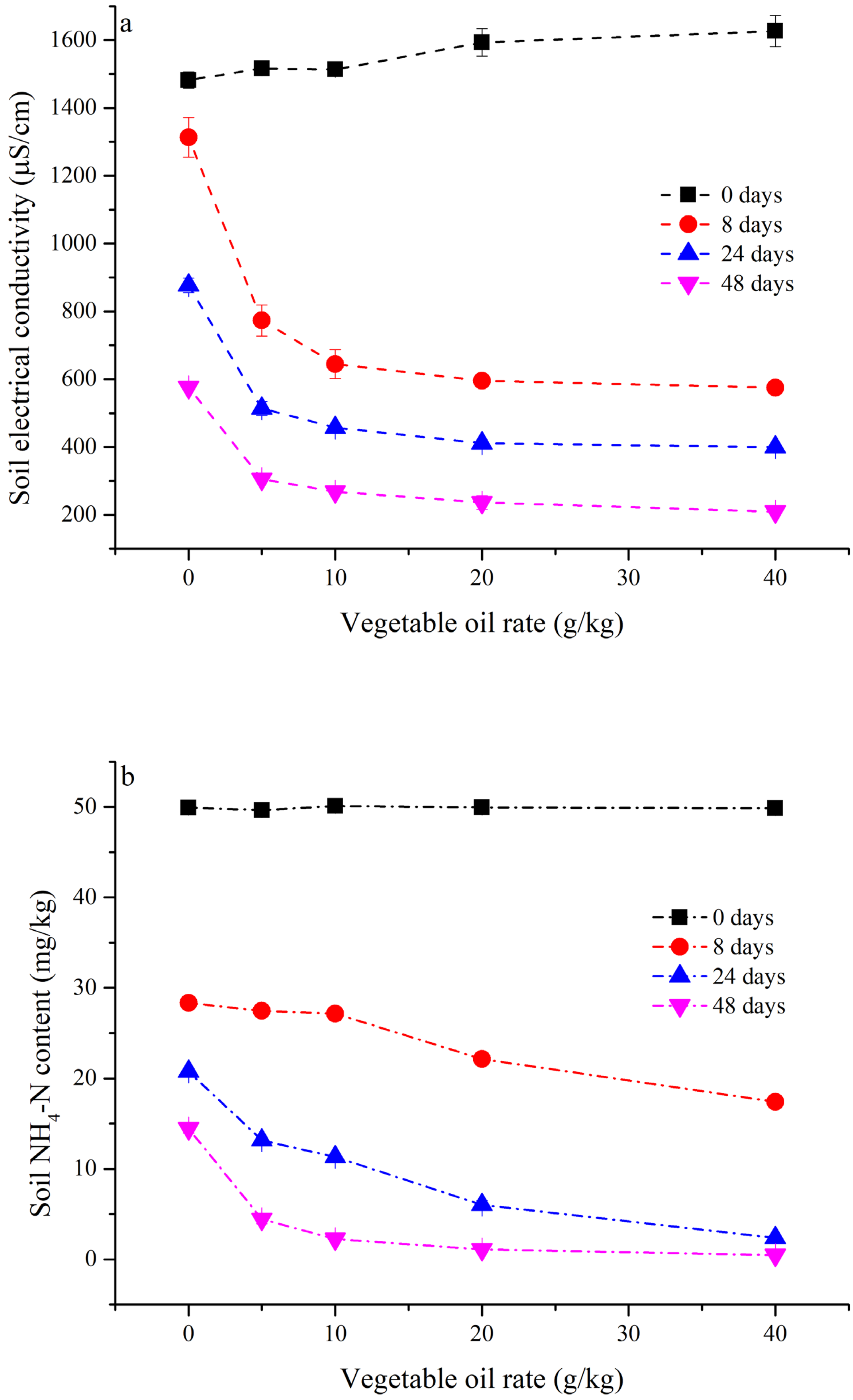
a
Soil electrical conductivity (μS/cm)
1600
1400
1200
1000
800
600
400
200
0 days
8 days
24 days
48 days
0
10
20
30
40
Vegetable oil rate (g/kg)
b
Soil NH4-N content (mg/kg)
50
40
30
20
10
0
0 days
8 days
24 days
48 days
0
10
20
30
40
Vegetable oil rate (g/kg)

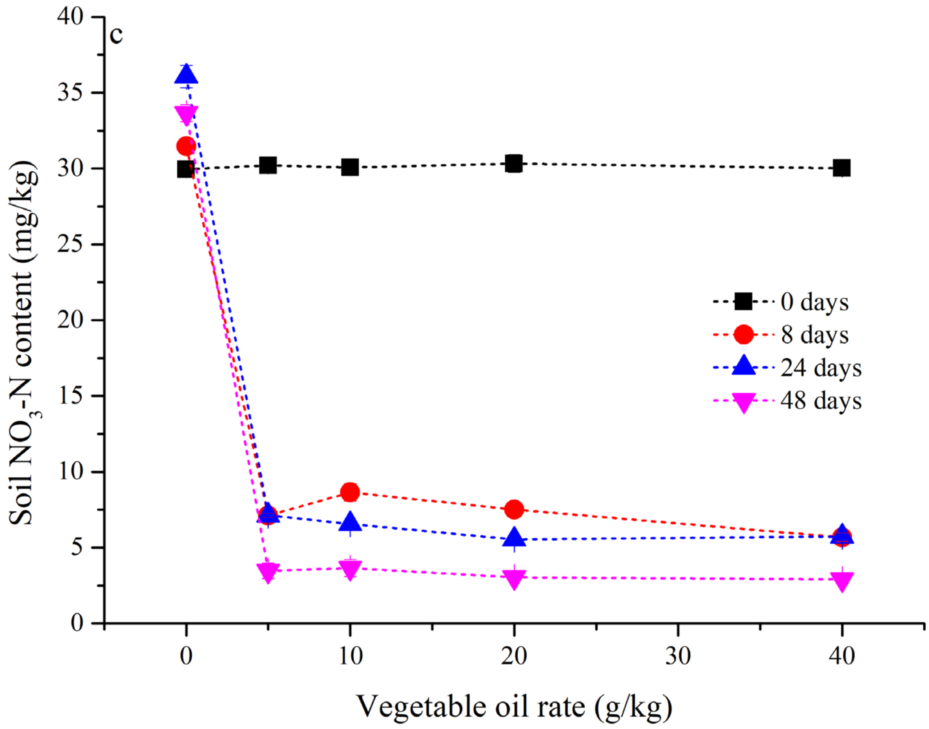
c
Soil NO_3-N content (mg/kg)
Vegetable oil rate (g/kg)
0 days
8 days
24 days
48 days

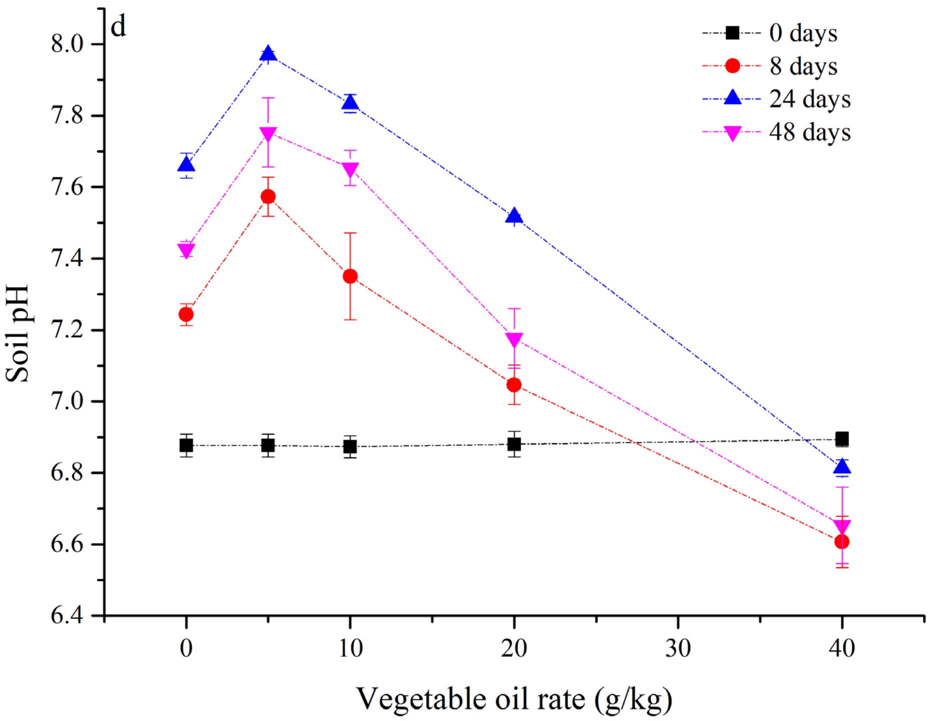
d
Soil pH
Vegetable oil rate (g/kg)
0 days
8 days
24 days
48 days

Figure S1. Trends of soil electrical conductivity (a), NH_4-N (b), NO_3-N (c) and pH (d) in the fertilizer and vegetable oil (FV-O) treatments during the mesocosm incubation (MI) experiment. Data shown are the means of three replicates.

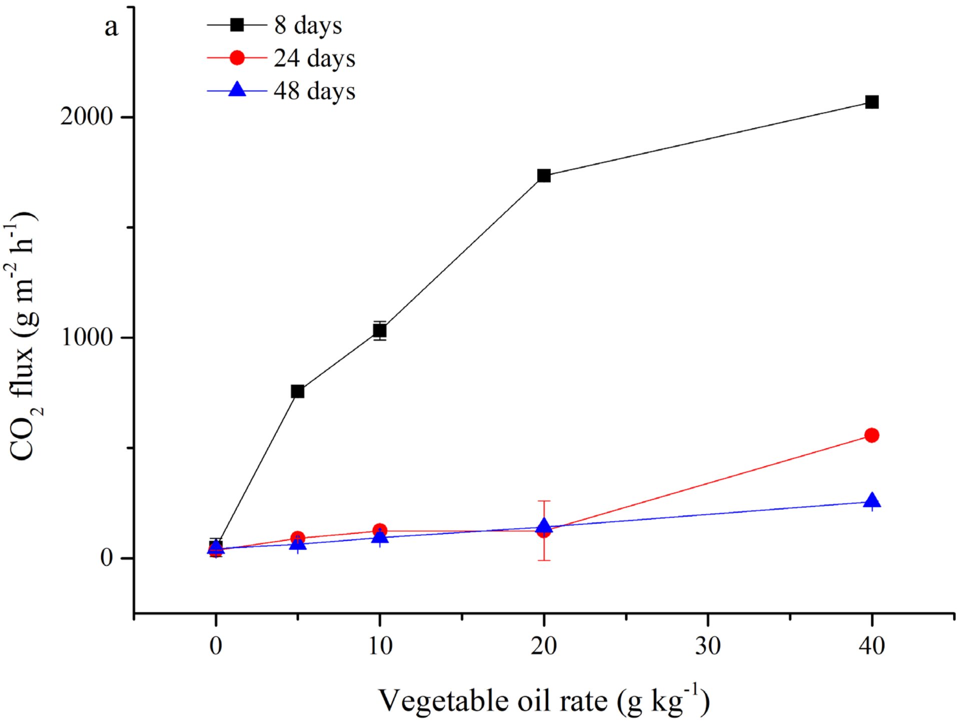
a
8 days
24 days
48 days
2000
1000
0
CO_2 flux (g m^{-2} h^{-1})
0
10
20
30
40
Vegetable oil rate (g kg^{-1})

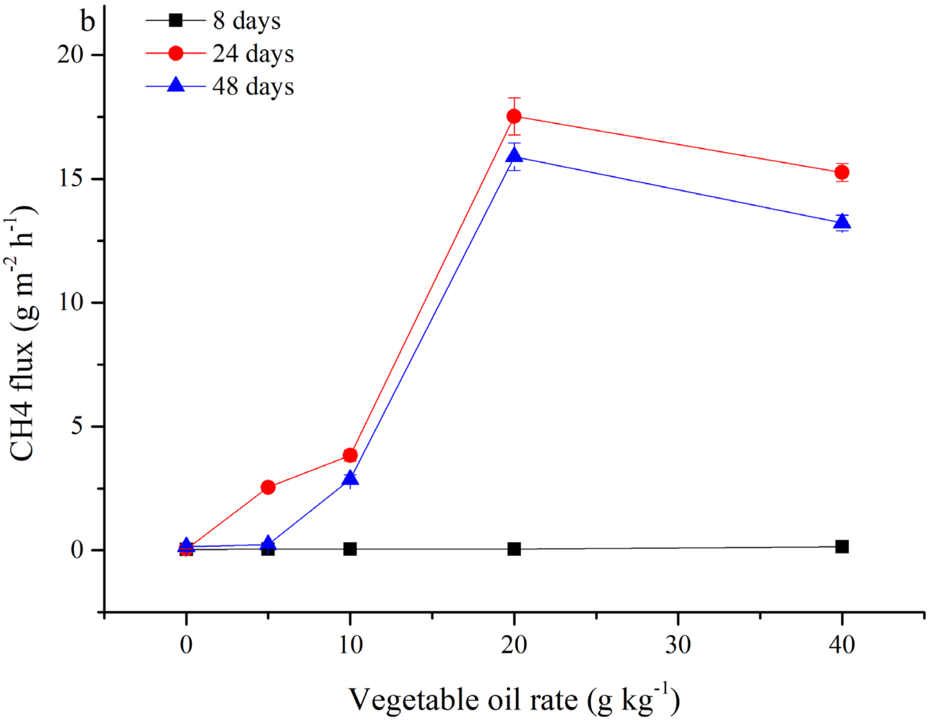
b
8 days
24 days
48 days
20
15
10
5
0
CH4 flux (g m^{-2} h^{-1})
0
10
20
30
40
Vegetable oil rate (g kg^{-1})

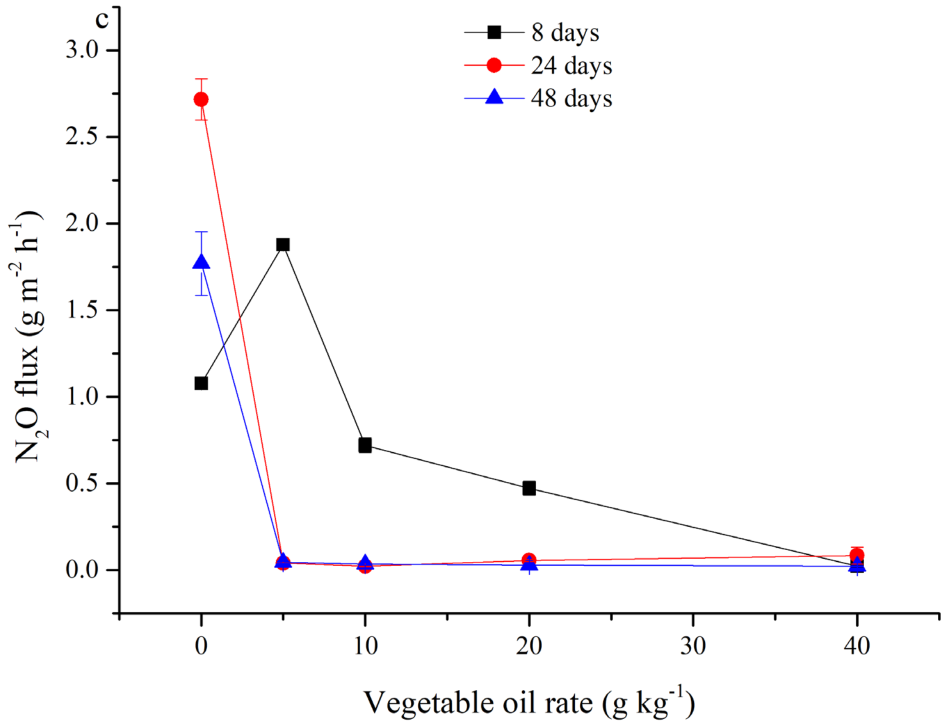

Figure S2. Soil greenhouse gas (GHG) fluxes in the fertilizer and vegetable oil (FV-O) treatments in mesocosm incubation (MI) experiment. (a) CO_2 flux, (b) CH_4 flux, and (c) N_2O flux.Data shown are the means of three replicates.

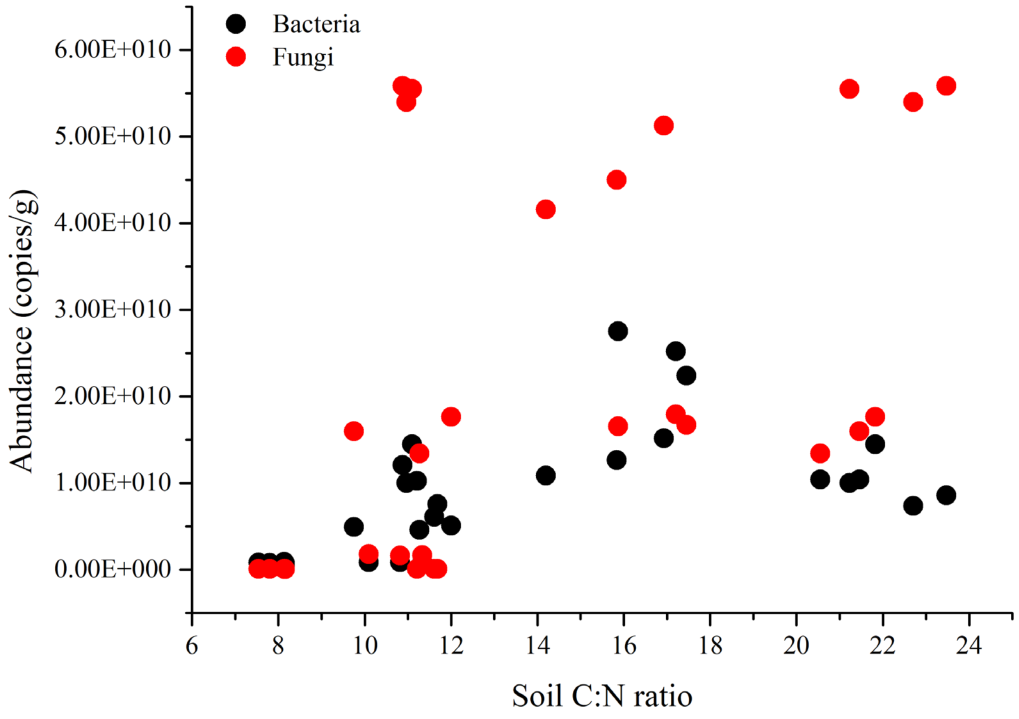

Figure S3. Responses of bacterial and fungal abundance to the soil C:N ratio in the mesocosm incubation (MI) experiment.

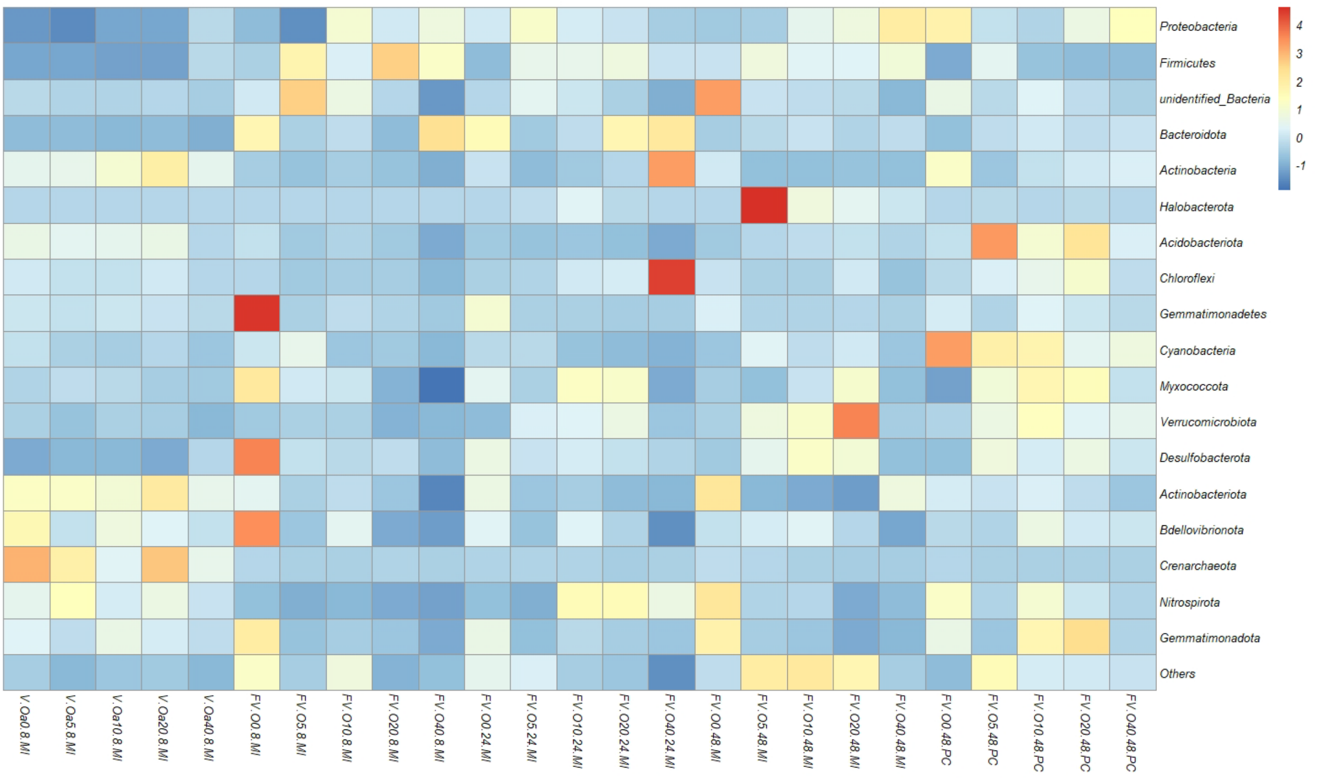

Figure S4. Bacterial communities at the phylum level for the treatments with the simultaneous application of fertilizer and vegetable oil (SIFVO) and the application of vegetable oil alone (VOa).V.Oa0.8.MI, V.Oa5.8.MI, V.Oa10.8.MI, V.Oa20.8.MI, and V.Oa40.8.MI represent VOa treatment with 0, 5, 10, 20, and 40 g/kg of vegetable oil on day 8 in the mesocosm incubation (MI) experiment, respectively. FV.O0.8.MI, FV.O5.8.MI, FV.O10.8.MI, FV.O20.8.MI, and FV.O40.8.MI represent SIFVO treatment with 0, 5,10, 20, and 40 g/kg of vegetable oil on day 8 in the MI experiment, respectively. FV.O0.24.MI, FV.O5.24.MI, FV.O10.24.MI, FV.O20.24.MI, and FV.O40.24.MI represent SIFVO treatment with 0, 5, 10, 20, and 40 g/kg of vegetable oil on day 24 in the MI experiment, respectively. FV.O0.48.MI, FV.O5.48.MI, FV.O10.48.MI, FV.O20.48.MI, and FV.O40.48.MI represent SIFVO treatment with 0, 5, 10, 20, and 40 g/kg of vegetable oil on day 48 in the MI experiment, respectively. FV.O0.48.MI, FV.O5.48.PC, FV.O10.48.PC, FV.O20.48.PC, and FV.O40.48.PC represent SIFVO treatment with 0, 5,10, 20, and 40 g/kg of vegetable oil on day 48 in the pot culture (PC) experiment respectively.

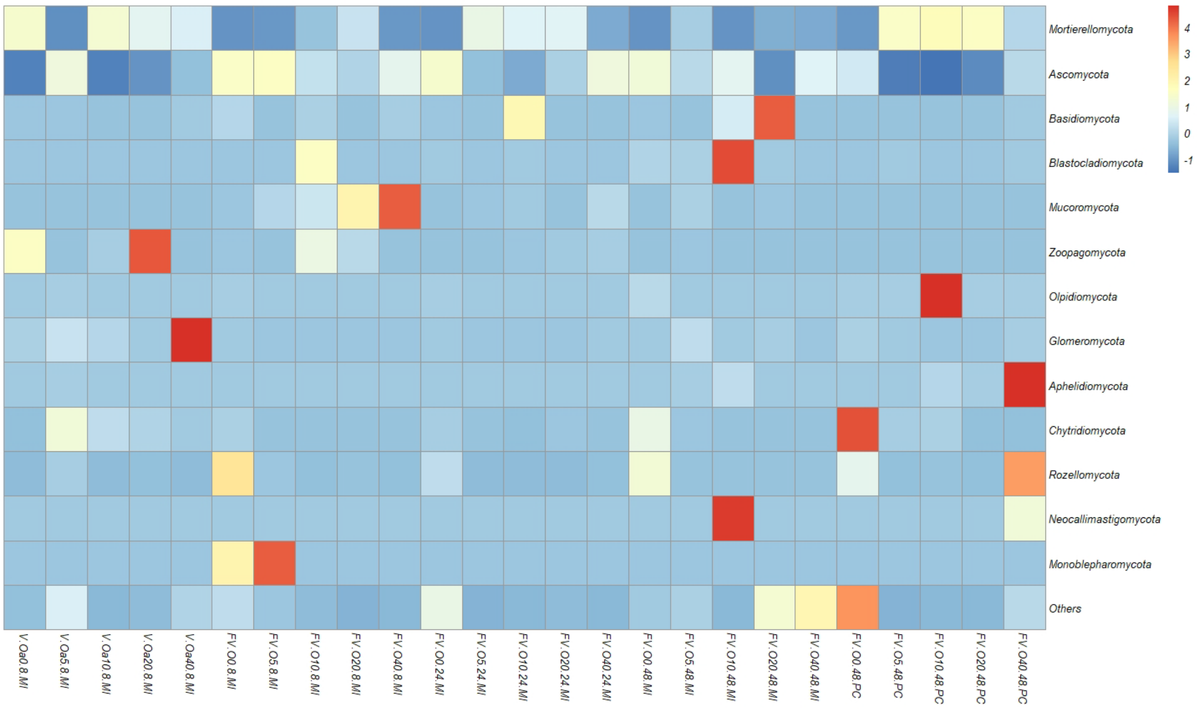

Figure S5. Fungal communities at the phylum level for the treatments with the simultaneous application of fertilizer and vegetable oil (SIFVO) and the application of vegetable oil alone (VOa).V.Oa0.8.MI, V.Oa5.8.MI, V.Oa10.8.MI, V.Oa20.8.MI, and V.Oa40.8.MI represent VOa treatment with 0, 5, 10, 20, and 40 g/kg of vegetable oil on day 8 in the mesocosm incubation (MI) experiment, respectively. FV.O0.8.MI, FV.O5.8.MI, FV.O10.8.MI, FV.O20.8.MI, and FV.O40.8.MI represent SIFVO treatment with 0, 5,10, 20, and 40 g/kg of vegetable oil on day 8 in the MI experiment, respectively. FV.O0.24.MI, FV.O5.24.MI, FV.O10.24.MI, FV.O20.24.MI, and FV.O40.24.MI represent SIFVO treatment with 0, 5, 10, 20, and 40 g/kg of vegetable oil on day 24 in the MI experiment, respectively. FV.O0.48.MI, FV.O5.48.MI, FV.O10.48.MI, FV.O20.48.MI, and FV.O40.48.MI represent SIFVO treatment with 0, 5, 10, 20, and 40 g/kg of vegetable oil on day 48 in the MI experiment, respectively. FV.O0.48.MI, FV.O5.48.PC, FV.O10.48.PC, FV.O20.48.PC, and FV.O40.48.PC represent SIFVO treatment with 0, 5,10, 20, and 40 g/kg of vegetable oil on day 48 in the pot culture (PC) experiment respectively.

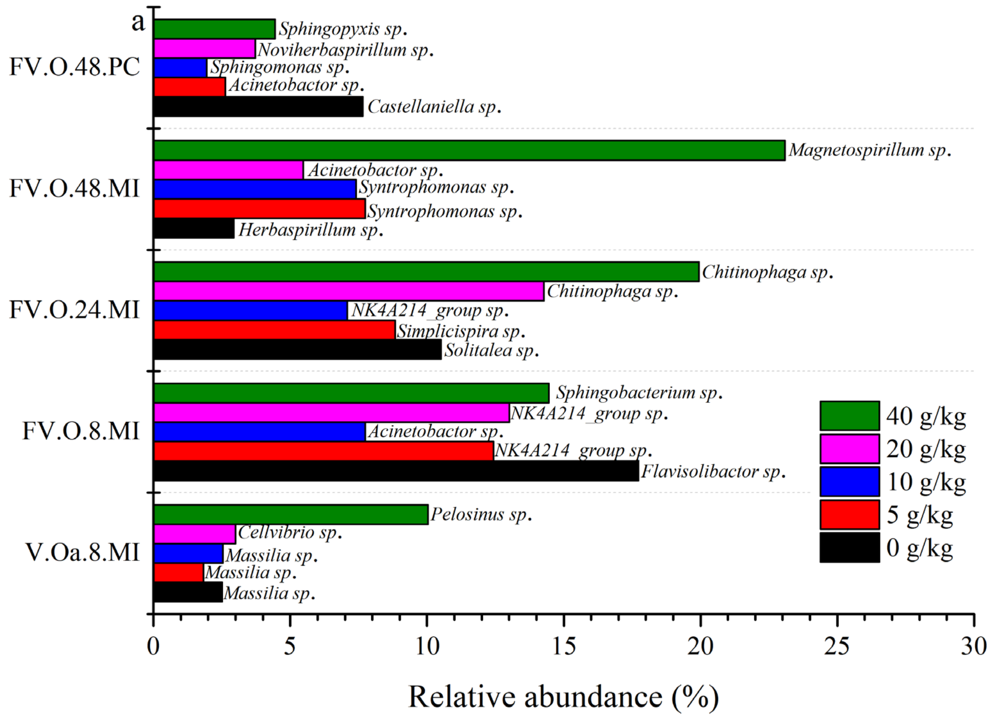
a
FV.O.48.PC
FV.O.48.MI
FV.O.24.MI
FV.O.8.MI
V.Oa.8.MI
Sphingopyxis sp.
Noviherbaspirillum sp.
Sphingomonas sp.
Acinetobactor sp.
Castellaniella sp.
Magnetospirillum sp.
Acinetobactor sp.
Syntrophomonas sp.
Syntrophomonas sp.
Herbaspirillum sp.
Chitinophaga sp.
Chitinophaga sp.
NK4A214_group sp.
Simplicispira sp.
Solitalea sp.
Sphingobacterium sp.
NK4A214_group sp.
Acinetobactor sp.
NK4A214_group sp.
Flavisolibactor sp.
Pelosinus sp.
Cellvibrio sp.
Massilia sp.
Massilia sp.
Massilia sp.
40 g/kg
20 g/kg
10 g/kg
5 g/kg
0 g/kg
0
5
10
15
20
25
30
Relative abundance (%)

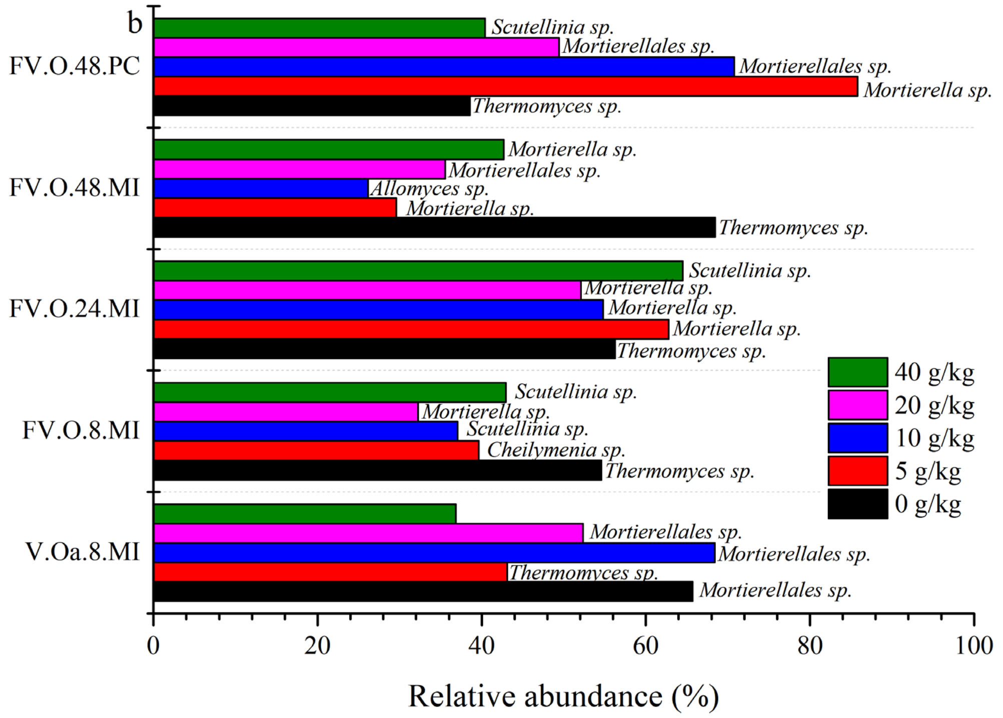
b
FV.O.48.PC
FV.O.48.MI
FV.O.24.MI
FV.O.8.MI
V.Oa.8.MI
Scutellinia sp.
Mortierellales sp.
Mortierellales sp.
Mortierella sp.
Thermomyces sp.
Mortierella sp.
Mortierellales sp.
Allomyces sp.
Mortierella sp.
Thermomyces sp.
Scutellinia sp.
Mortierella sp.
Mortierella sp.
Mortierella sp.
Thermomyces sp.
Scutellinia sp.
Mortierella sp.
Scutellinia sp.
Cheilymenia sp.
Thermomyces sp.
Mortierellales sp.
Mortierellales sp.
Thermomyces sp.
Mortierellales sp.
40 g/kg
20 g/kg
10 g/kg
5 g/kg
0 g/kg
0
20
40
60
80
100
Relative abundance (%)

Figure S6. Relative abundances of the top bacterial genera (a) and the top fungal genera (b) under different vegetable oil application rate.V.Oa.8.MI, FV.O.8.MI, FV.O.24.MI, FV.O.48.MI, and FV.O.48.PC represent the treatments with vegetable oil application alone on day 8 in the mesocosm incubation (MI) experiment. FV.O.8.MI, FV.O.24.MI, and FV.O.48.MI represent the simultaneous application of fertilizer and vegetable oil treatments on days 8, 24, and 48 in the MI experiment, respectively. FV.O.48.PC represents the simultaneous application of fertilizer and vegetable oil treatments on day 48 in the pot culture (PC) experiment.

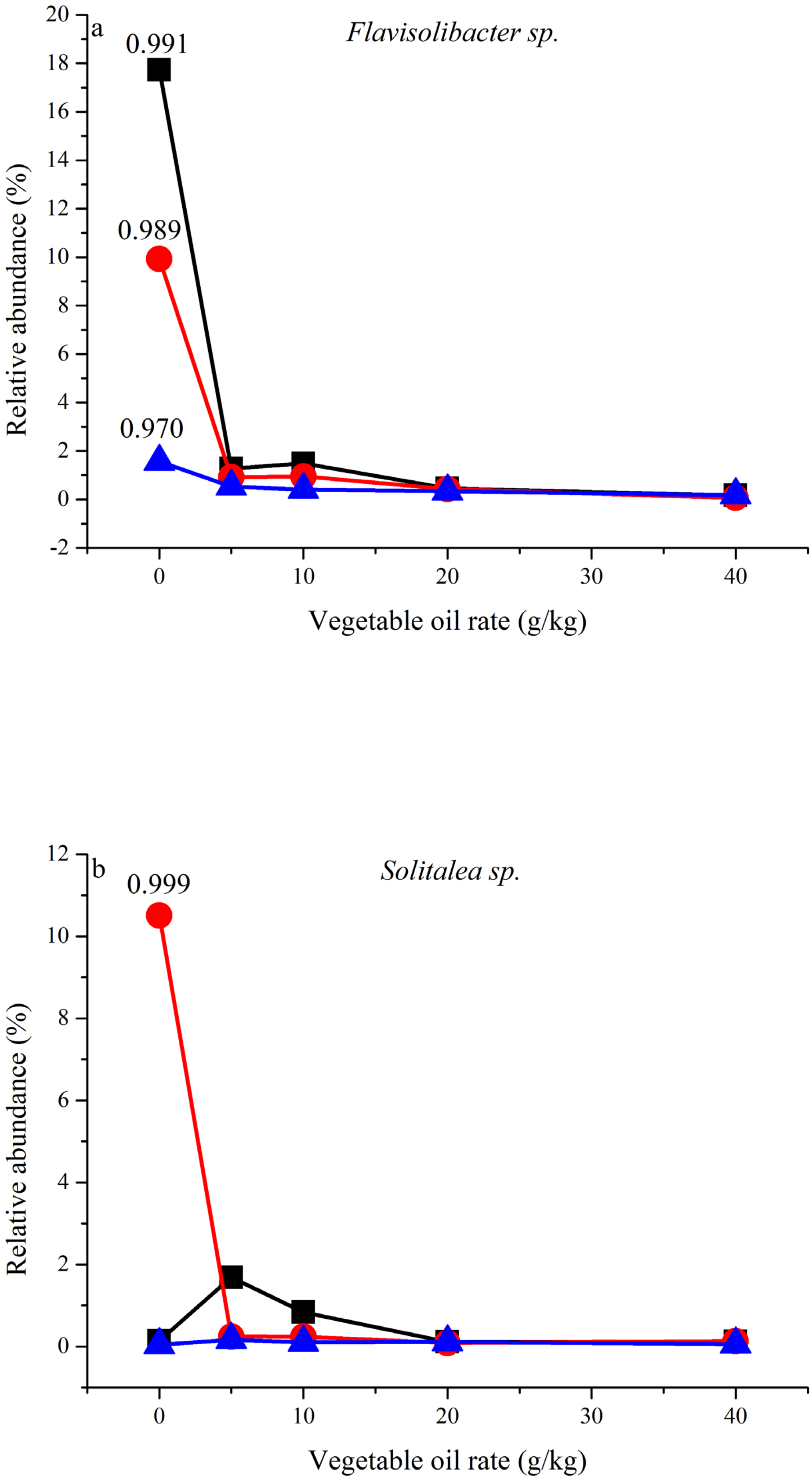
a
Flavisolibacter sp.
0.991
0.989
0.970
Relative abundance (%)
Vegetable oil rate (g/kg)
b
Solitalea sp.
0.999
Relative abundance (%)
Vegetable oil rate (g/kg)

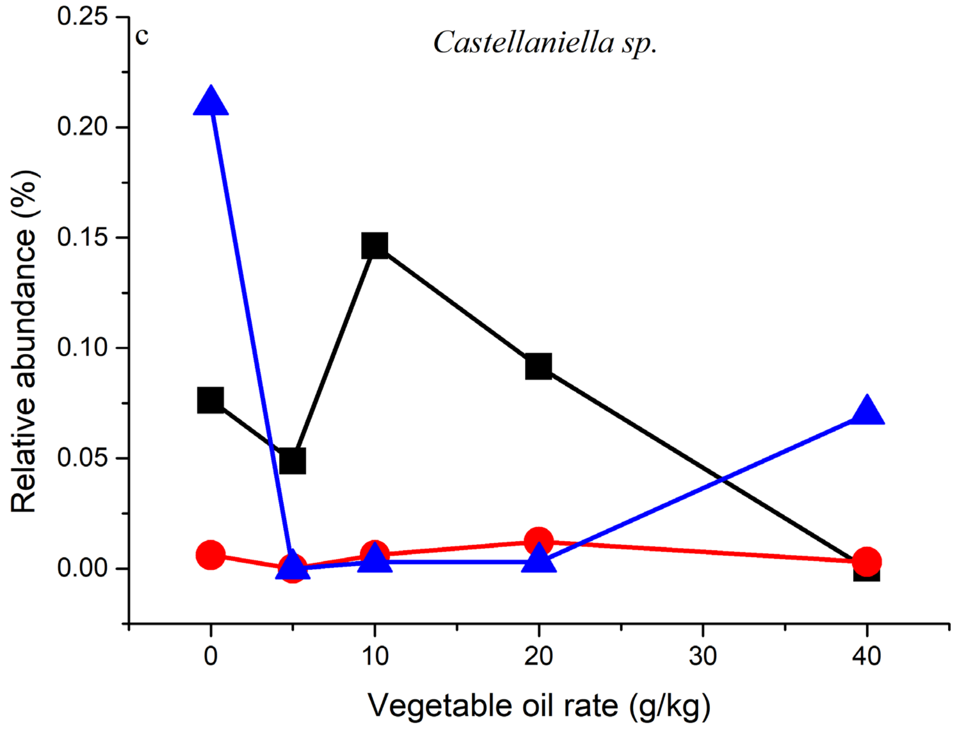
c
Castellaniella sp.
Relative abundance (%)
0.25
0.20
0.15
0.10
0.05
0.00
0
10
20
30
40
Vegetable oil rate (g/kg)

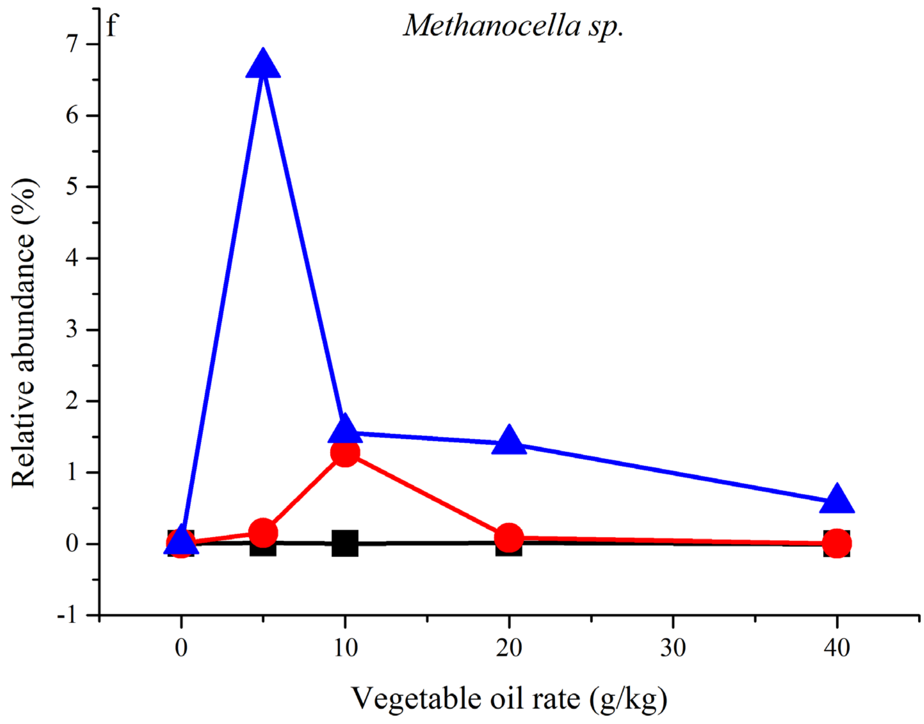
f
Methanocella sp.
Relative abundance (%)
7
6
5
4
3
2
1
0
-1
0
10
20
30
40
Vegetable oil rate (g/kg)

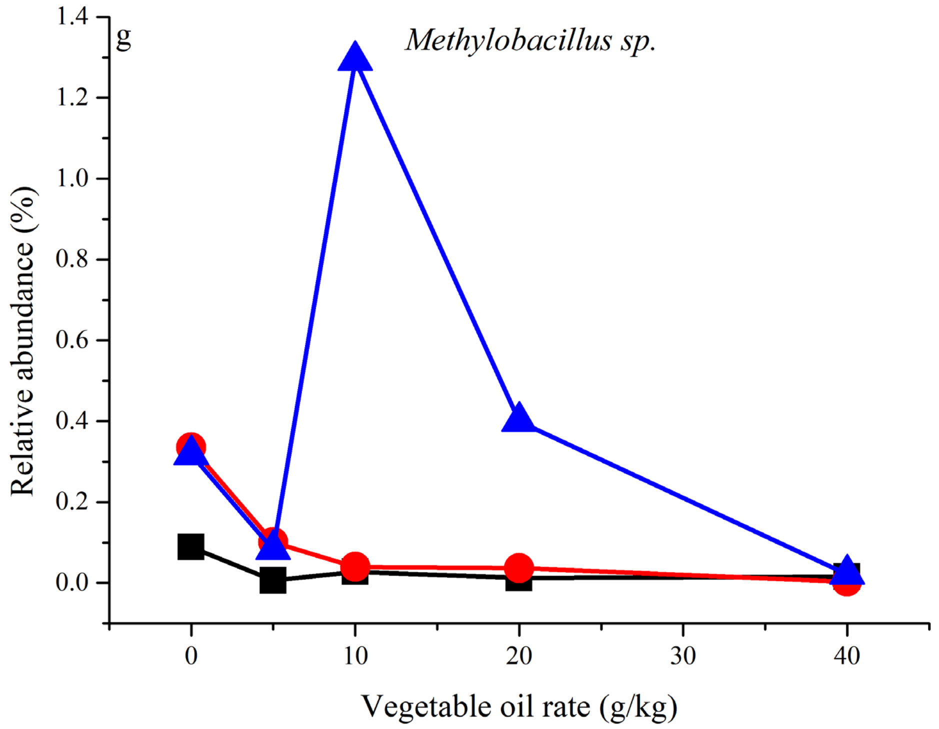

g
Methylobacillus sp.
Relative abundance (%)
Vegetable oil rate (g/kg)

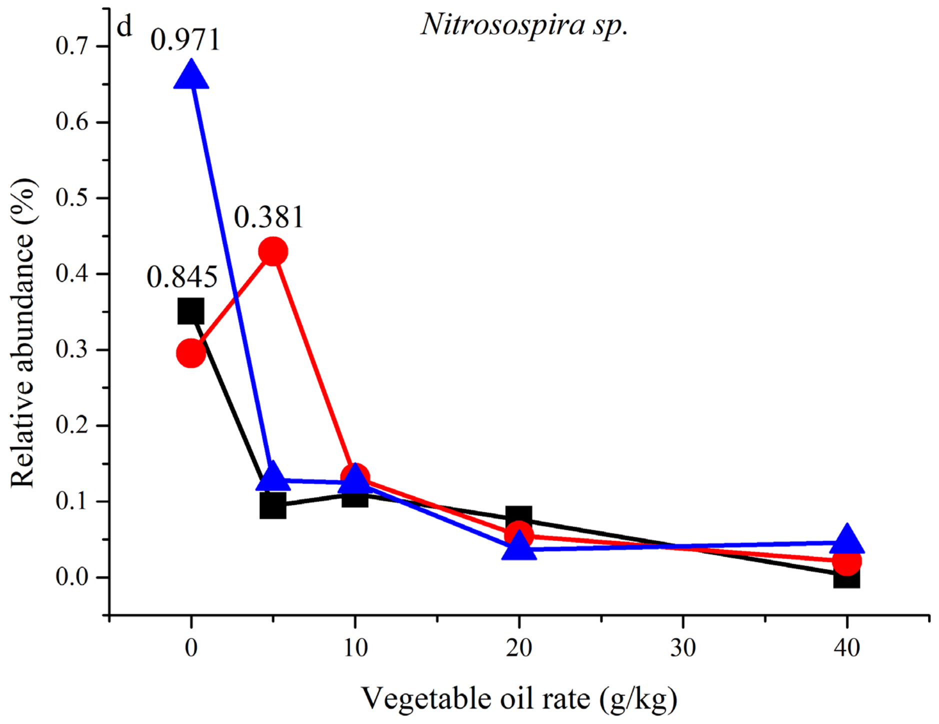

d
Nitrosospira sp.
0.971
0.381
0.845
Relative abundance (%)
Vegetable oil rate (g/kg)

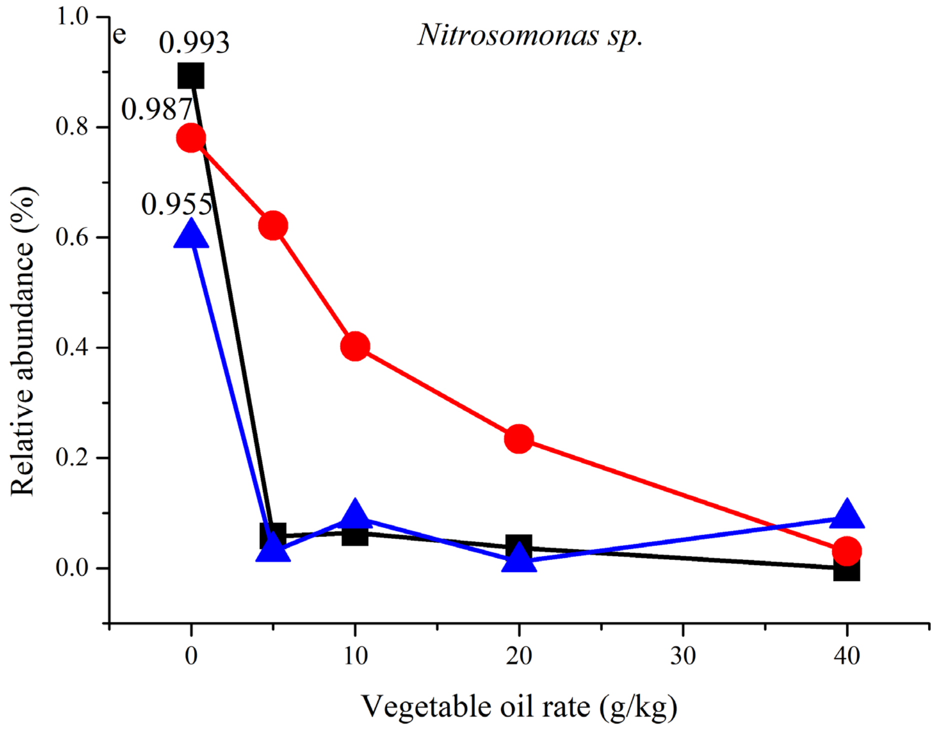
e
Nitrosomonas sp.
0.993
0.987
0.955
Relative abundance (%)
Vegetable oil rate (g/kg)
1.0
0.8
0.6
0.4
0.2
0.0
0
10
20
30
40

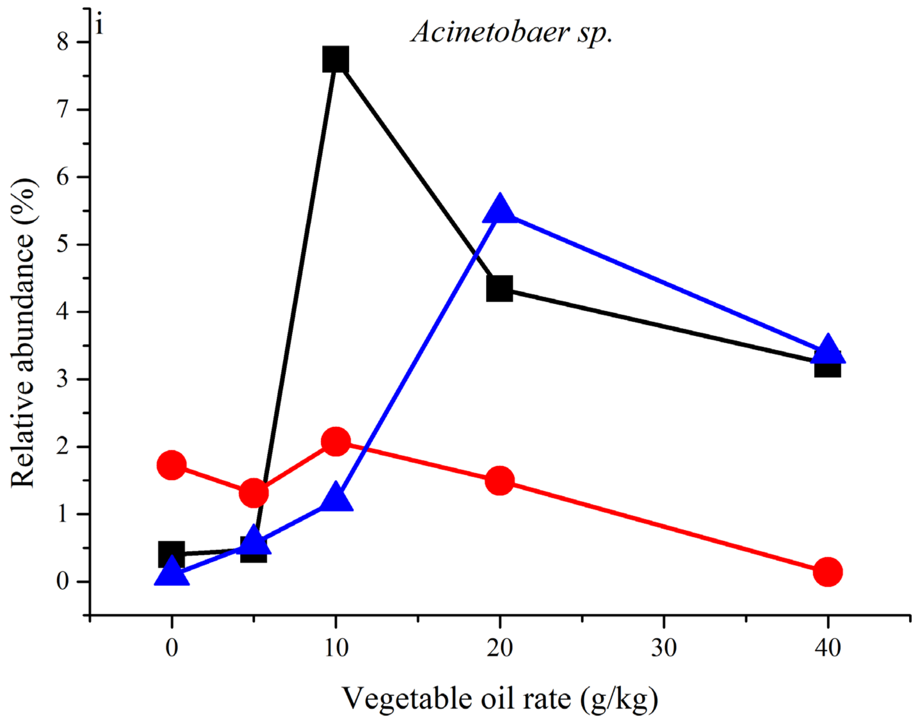
i
Acinetobaer sp.
Relative abundance (%)
Vegetable oil rate (g/kg)
8
7
6
5
4
3
2
1
0
0
10
20
30
40

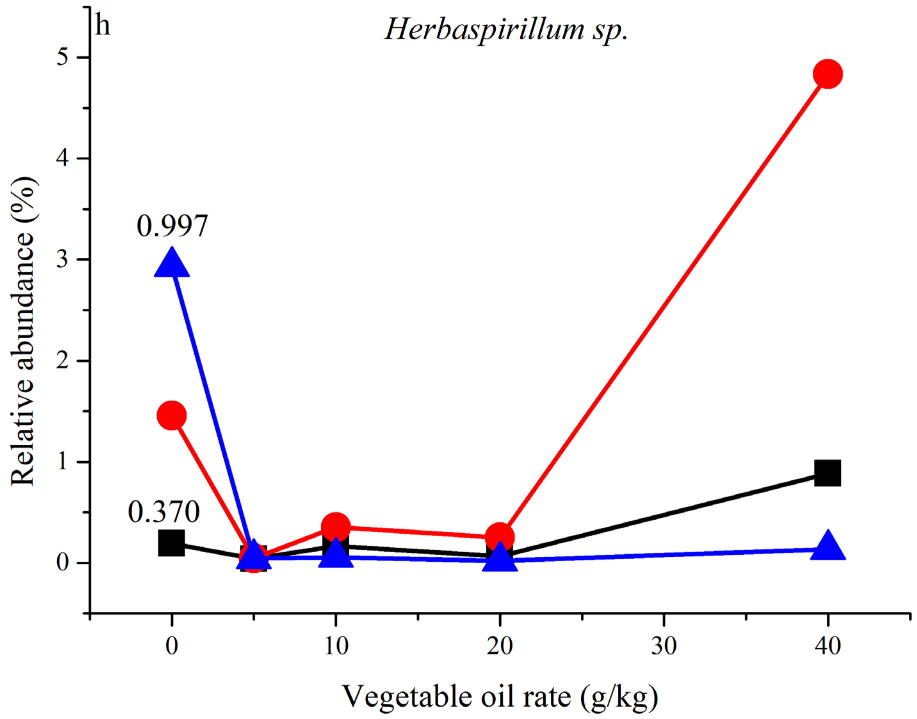
h
Herbaspirillum sp.
0.997
0.370
Relative abundance (%)
Vegetable oil rate (g/kg)
0
1
2
3
4
5
0
10
20
30
40

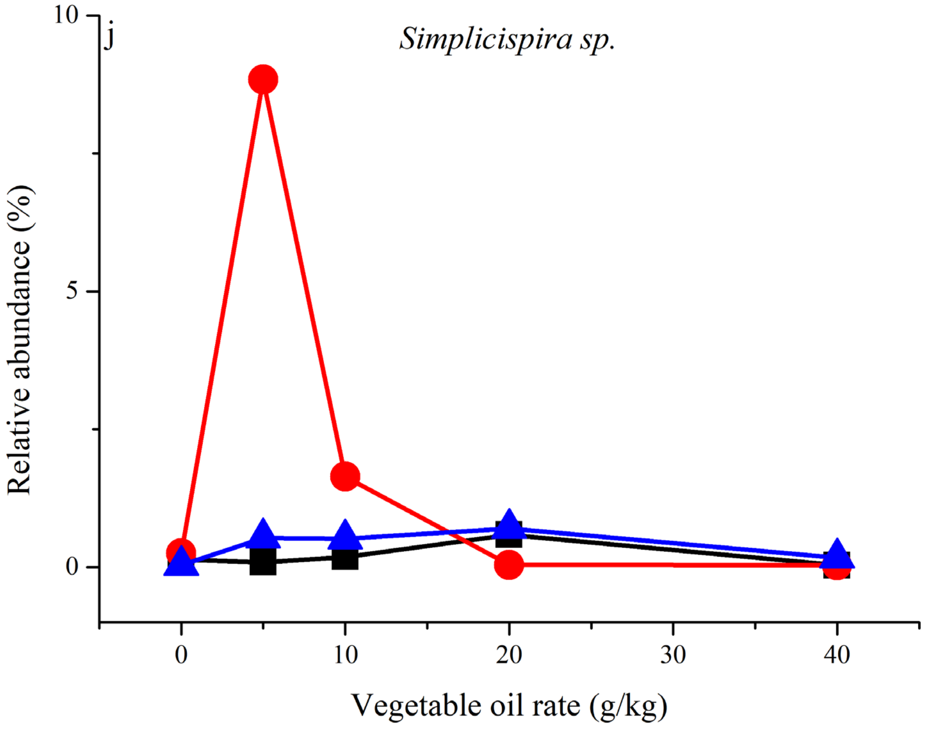
j
Simplicispira sp.
Relative abundance (%)
Vegetable oil rate (g/kg)
0
5
10
0
10
20
30
40

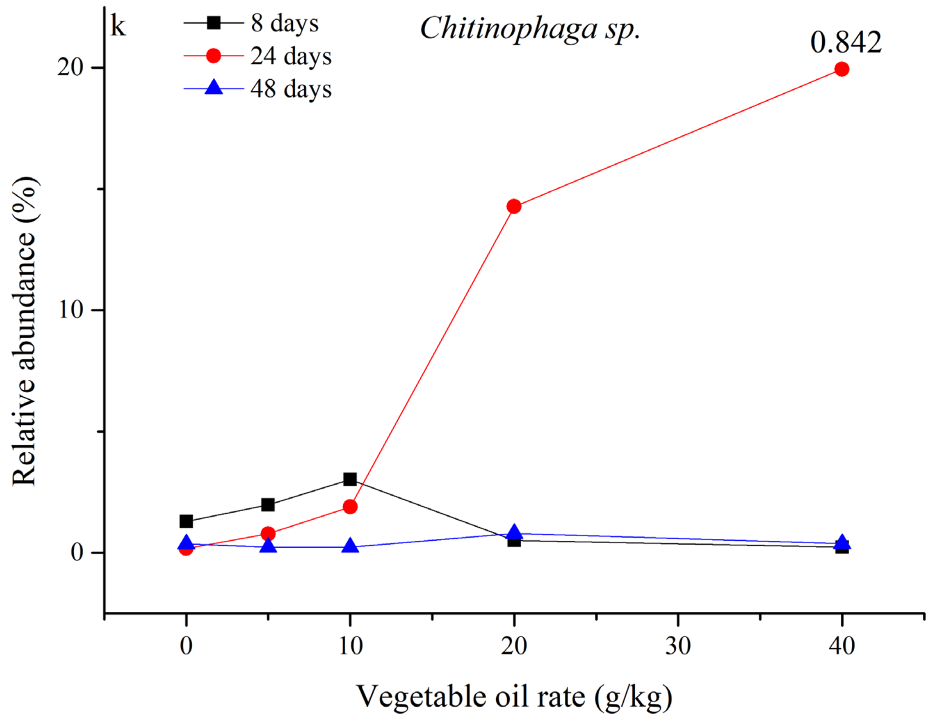

Figure S7. Responses of the relative abundances of key bacterial taxa to the vegetable oil application rate. The number labeled on the symbol represents the coefficient of determination (R^2) in the fitted exponential equation. The unlabeled curves were insignificant in fitting the relationship between the relative abundance and the vegetable oil application rate.

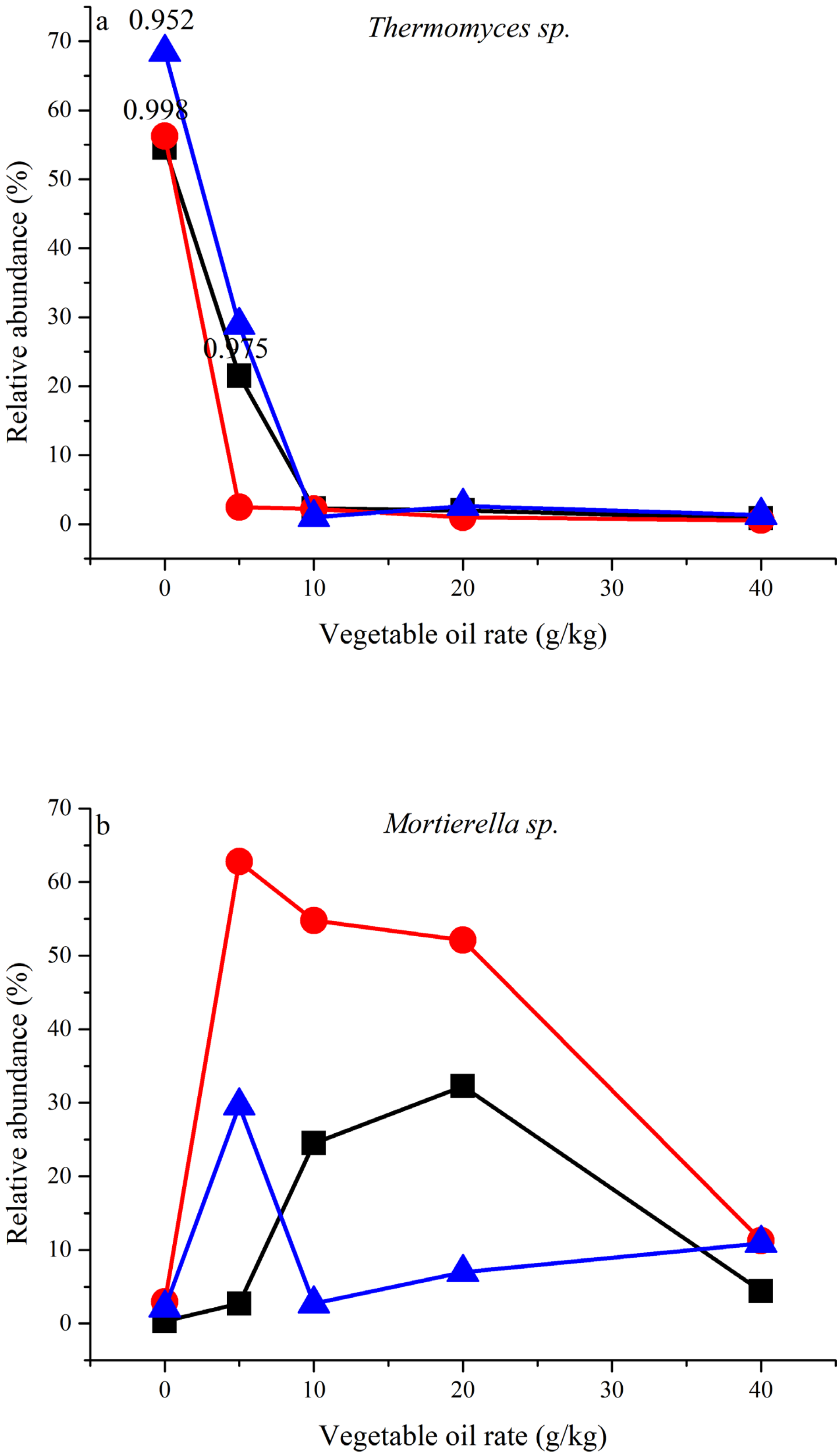

a
Thermomyces sp.
0.952
0.998
0.975
Relative abundance (%)
Vegetable oil rate (g/kg)
b
Mortierella sp.
Relative abundance (%)
Vegetable oil rate (g/kg)

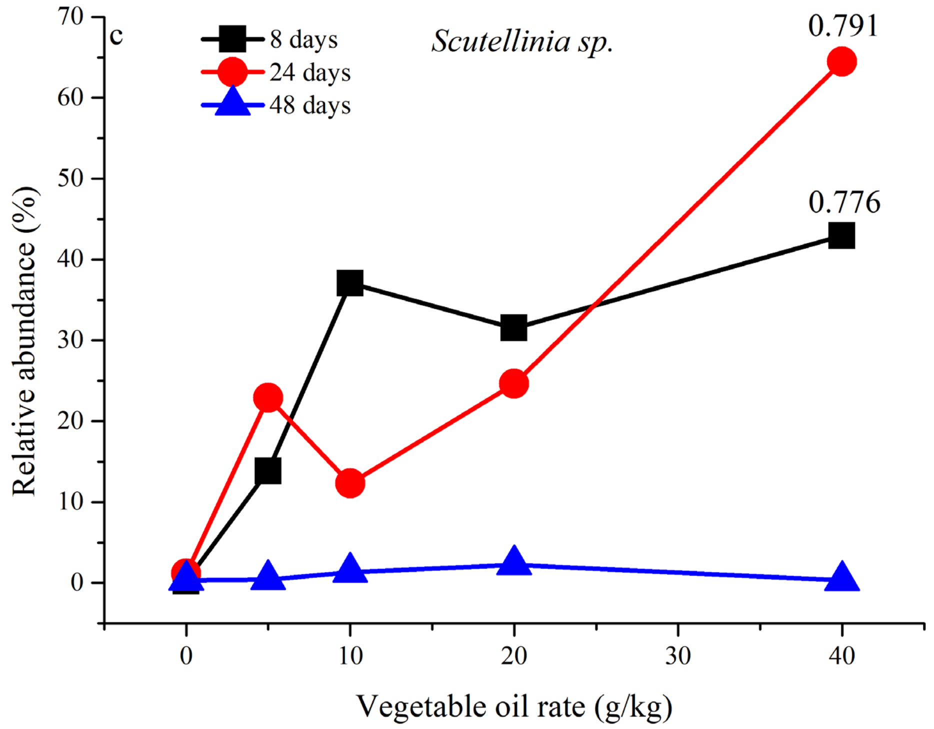

Figure S8. Responses of the relative abundances of key fungal taxa to vegetable oil application rates. The number labeled on the symbol represents the coefficient of determination (R^2) in the fitted exponential equation. The unlabeled curves were insignificant in fitting the relationship between the relative abundance and the vegetable oil application rate.

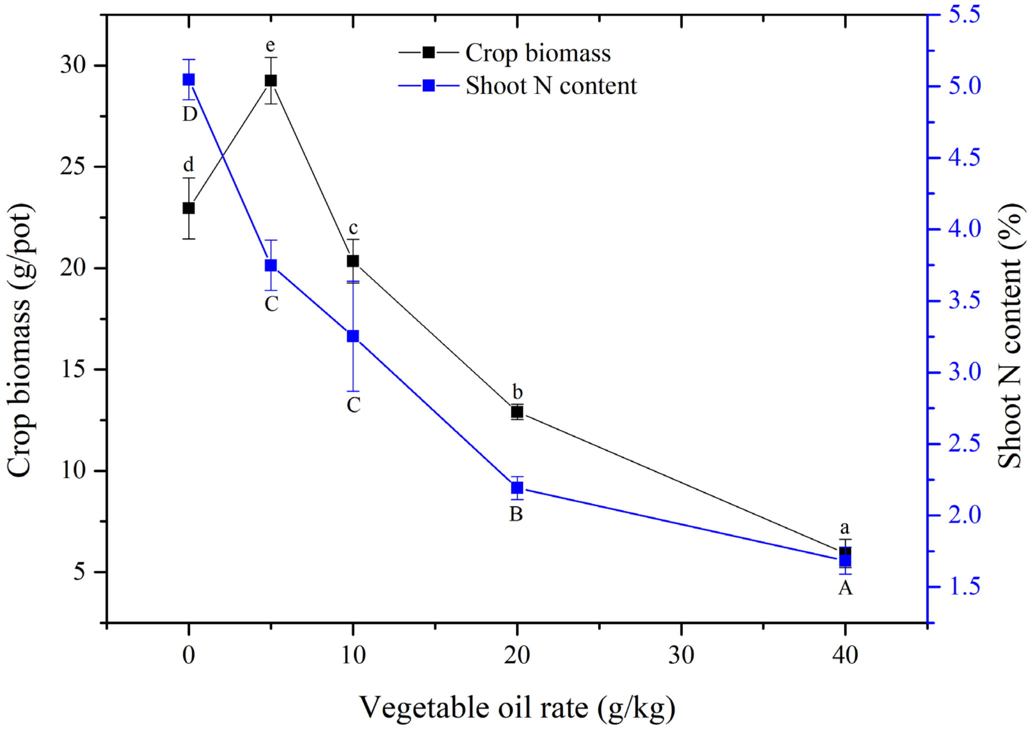

Figure S9. Crop biomass and the shoot N content under different vegetable oil application rates in the pot culture (PC) experiment.Data shown are the means of three replicates, and different letter indicates significant at 0.05.
